# Identification of Predominant Histopathological Growth Patterns of Colorectal Liver Metastasis by Multi-Habitat and Multi-Sequence Based Radiomics Analysis

**DOI:** 10.3389/fonc.2020.01363

**Published:** 2020-08-14

**Authors:** Yuqi Han, Fan Chai, Jingwei Wei, Yali Yue, Jin Cheng, Dongsheng Gu, Yinli Zhang, Tong Tong, Weiqi Sheng, Nan Hong, Yingjiang Ye, Yi Wang, Jie Tian

**Affiliations:** ^1^School of Life Science and Technology, Xidian University, Xi'an, China; ^2^Key Laboratory of Molecular Imaging, Institute of Automation, Chinese Academy of Sciences, Beijing, China; ^3^Beijing Key Laboratory of Molecular Imaging, Beijing, China; ^4^Department of Radiology, Peking University People's Hospital, Beijing, China; ^5^Department of Radiology, Fudan University Shanghai Cancer Center, Shanghai, China; ^6^Department of Oncology, Shanghai Medical College, Fudan University, Shanghai, China; ^7^Department of Pathology, Peking University People's Hospital, Beijing, China; ^8^Department of Pathology, Fudan University Shanghai Cancer Center, Shanghai, China; ^9^Department of Gastrointestinal Surgery, Peking University People' Hospital, Beijing, China; ^10^Beijing Advanced Innovation Centre for Big Data-Based Precision Medicine, School of Medicine, Beihang University, Beijing, China; ^11^Engineering Research Centre of Molecular and Neuro Imaging of Ministry of Education, School of Life Science and Technology, Xidian University, Xi'an, China

**Keywords:** colorectal cancer, liver metastasis, magnetic resonance, histopathologic growth patterns, radiomics

## Abstract

**Purpose:** Developing an MRI-based radiomics model to effectively and accurately predict the predominant histopathologic growth patterns (HGPs) of colorectal liver metastases (CRLMs).

**Materials and Methods:** In this study, 182 resected and histopathological proven CRLMs of chemotherapy-naive patients from two institutions, including 123 replacement CRLMs and 59 desmoplastic CRLMs, were retrospectively analyzed. Radiomics analysis was performed on two regions of interest (ROI), the tumor zone and the tumor-liver interface (TLI) zone. Decision tree (DT) algorithm was used for radiomics modeling on each MR sequence, and fused radiomics model was constructed by combining the radiomics signature of each sequence. The clinical and combination models were developed through multivariate logistic regression method. The performance of the developed models was assessed by receiver operating characteristic (ROC) curves with indicators of area under curve (AUC), accuracy, sensitivity, and specificity. A nomogram was constructed to evaluate the discrimination, calibration, and usefulness.

**Results:** The fused radiomics^tumor^ and radiomics^TLI^ models showed better performance than any single sequence and clinical model. In addition, the radiomics^TLI^ model exhibited better performance than radiomics^tumor^ model (AUC of 0.912 vs. 0.879) in internal validation cohort. The combination model showed good discrimination, and the AUC of nomogram was 0.971, 0.909, and 0.905 in the training, internal validation, and external validation cohorts, respectively.

**Conclusion:** MRI-based radiomics method has high potential in predicting the predominant HGPs of CRLM. Preoperative non-invasive identification of predominant HGPs could further explore the ability of HGPs as a potential biomarker for clinical treatment strategy, reflecting different biological pathways.

## Introduction

The inter- and intra-lesion heterogeneities of genetic, epigenetic, phenotypic, and morphologic characteristics leads to differences in overall survival response to systemic treatment in patients with colorectal liver metastasis (CRLM) (1). One of these heterogeneities appears as histopathological growth patterns (HGPs) and corresponding microvasculatures (2, 3). According to the different interface between tumor cells and adjacent liver parenchyma, CRLM mainly has two types of HGPs: the desmoplastic and the replacement; other infrequent types include the pushing and mixed HGPs (4). In the desmoplastic HGP, tumor cells and liver parenchyma are separated by a fibrous rim with lymphocytic infiltration and have a microvasculature of sprouting angiogenesis (3). In the replacement HGP, the tumor cells forming the cell plate are continuous with the hepatocyte plate, with microvasculature of vessel co-option and without angiogenesis (3). The diversity of these biological microenvironments leads to different responses to treatment, especially to the anti-angiogeneic agent (5, 6) and to different long-term prognosis (3, 7, 8). The replacement HGP is identified as a poor predictor of Bevacizumab treatment in patients with CRLMs (9).

The gold standard for HGP diagnosis of CRLMs is the histopathological analysis of the chemo-naïve resected specimen (3). Due to the low percentage of initially resectable lesions and the wide use of preoperative systemic treatment, clinical relevance is seriously limited. Therefore, a preoperative and non-invasive surrogate method is needed to predict different HGP in CRLMs, so as to improve the prognosis and facilitate the treatment strategy. In addition, a non-invasive method to assess the HGPs would also allow for longitudinal follow-up of the response of a lesion to a certain treatment by switch of the HGP.

Heightened soft-tissue resolution, multiparameter acquisition, and functional imaging enable the MRI to be an invaluable imaging method for patients with CRLMs (10). Although there is no direct evidence that the qualitative and quantitative MRI features can predict HGP of CRLMs, preliminary data have provided some clues. In a very limited cohort of seven patients with liver metastases, Semelka et al. demonstrated that the transient enhancement around the lesion on MR images was related to the desmoplastic reaction, inflammatory cell infiltration, and vascular proliferation around the tumor (11). Based on dynamic contrast enhanced (DCE) MRI, O'Connor and Jayson revealed the microvascular heterogeneity is a prognostic and predictive biomarker before bevacizumab containing therapy (12). However, dedicated acquisition and analysis protocol of DCE-MRI also limits clinical relevance (13).

Compared with qualitative gross imaging features, radiomics transforms digital images into quantitative data, analyzes the spatial heterogeneity, and generates imaging biomarkers that can be used as an assistant tool for clinical decision-making (14). It develops rapidly in cancer detection, diagnosis, and therapeutic strategy selection, prediction of prognosis, therapeutic response, and surveillance (15–18). Our previous study showed multidetector CT based radiomics analysis could effectively identify HGP of CRLMs (19). Nevertheless, as far as we know, radiomics model for predicting HGP of CRLMs based on MR images has not been established.

In this study, we aim to develop and validate an MRI-based radiomics model for predicting HGP of CRLMs, so as to effectively screen patients and develop appropriate treatment strategies.

## Materials and Methods

**Figure 1:**
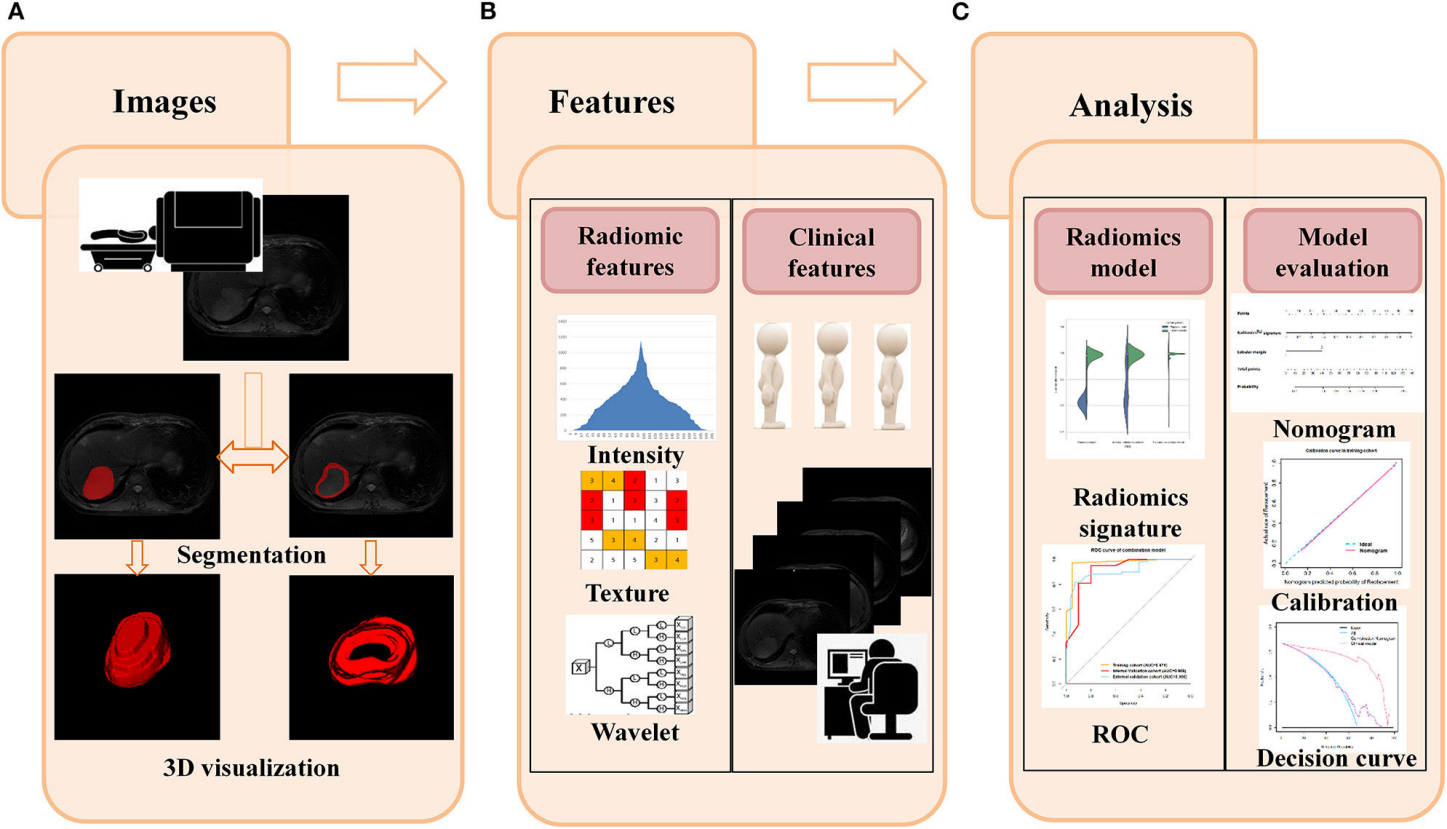
Flowchart for this study. **(A)** The T2W images were used as an example to illustrate the segmentation process, and slicer-to-slicer delineation obtained two sets of ROI. **(B)** Multiple radiomic features were extracted from two sets of ROI. Clinical features were obtained from medical records, and qualitative gross imaging features were evaluated by two board-certified radiologists. **(C)** Intra-/inter-observer coefficients were used to select the stability features, and decision tree were used to construct the radiomics model in each sequence. The best performed sequences and related clinical features were used to form the final classification model. The ROC, calibration curve, and decision curve were used to evaluate the performance of models.

Two institutional review boards supported this study and waived the informed consent requirements due to retrospective analysis. The flowchart of this study is showed in Figure 1.

### Patient Population

Patients were retrospectively searched in both institutions' histopathological electronic information systems (HIS) from November 2007 and June 2018. The inclusion criteria were as follows: (1) partial hepatectomy was performed; (2) CRLM was proved by histopathological analysis; and (3) a contrast-enhanced abdominal MRI examination was performed within 4 weeks before surgery. The exclusion criteria were as follows: (1) pre-operative systemic and/or regional treatments were performed; (2) MR image quality was inadequate for analysis; and (3) hematoxylin- and eosin-stained (H&E) sections of the tumor-liver interface (TLI) areas of the resected CRLM specimen were inadequate for analysis.

Several clinical characteristics, including age, sex, classification of the CRLMs (synchronous vs. metachronous), time interval from MR scanning to hepatectomy, number of resected lesions per patient, location of primary lesions (left-sided: from the splenic flexure to the rectum vs. right-sided: from the ileocecal junction to the transverse colon) (20), histopathological type and tumor differentiation (high-/moderate- vs. low-differentiated adenocarcinoma) of the primary lesion were derived.

### Histopathological Analysis

For all resected specimens, two experienced pathologists (YLZ, 10 years of experience; WQS, 15 years of experience), who were ignorant to the clinical data, reviewed the H&E-stained section from four formalin-fixed paraffin-embedded blocks according to the international consensus guidelines (3). Any subjective disagreement between the two readers was discussed until consensus was reached.

Lesions were categorized according to the 50% cut-off value of the consensus guidelines. Lesions were categorized as desmoplastic, replacement, or pushing HGP when >50% of the interface was scored (i.e., >50% desmoplastic appearance would be classified into desmoplastic HGP; same as the replacement and the pushing HGP). Lesion was considered to be mixed HGP if none of the three HGP was present at >50% of the interface.

### MRI Acquisition and Qualitative Feature Analysis

#### MRI Acquisition

The MRI examinations were performed by 750 W system (GE Healthcare, Milwaukee, WI, USA) and 8-channel phased array torso coils in both institutions. All patients were positioned supine and feet-first. Images used in this study included (i) T2-weighted image (T2WI) of fast recovery fast spin-echo with fat saturation, (ii) Diffusion-weighted imaging and the Apparent diffusion coefficient (ADC) quantification, (iii) T1-weighted image (T1WI), (iv) arterial phase (AP), and (v) portal venous phase (PVP) image of the dynamic contrast-enhanced T1-weighted liver acquisition volume acceleration (LAVA) with chemically selective fat saturation. The conventional abdominal MRI protocol is shown in Table E1. The enhanced images were acquired after the intravenous administration of gadopentetate dimeglumine (Magnevist; Bayer HealthCare Pharmaceuticals, Berlin, Germany) in the arterial phase (46 s after contrast injection) and portal venous phases (79 s after contrast injection).

#### Qualitative Feature Analysis

Two board-certified abdominal radiologists (FC, 2 years of experience; JC, 10 years of experience), who were ignorant to the histopathologic information and the original MR imaging reports, evaluated the MR image retrospectively and independently. Any subjective disagreement between the two readers was discussed until consensus was reached.

The following features were evaluated and recorded: (1) the location of the tumor: segment I–IV vs. segment V–VIII, based on the Couinaud criteria (21); (2) the contour of the tumor: lobular (defined as a tumor with one or more indentation with an acute angle) or non-lobular (no indentations with an acute angle) (22); (3) the presence or absence of enhanced rim on AP or PVP appearing as a peritumoral, complete ring with higher intensity relative to adjacent liver parenchyma, as well as the solid component of the lesion (11); and (4) the maximum diameter of the tumor (in millimeters) in the axial PVP images.

### Model Construction and Assessment

All of these characteristics were further investigated by developing of three models, specifically (1) a radiomics model, including the selected radiomic features; (2) a clinical model, including the clinical and qualitative imaging features; and (3) a combination model, including the selected clinical, qualitative imaging, and radiomic features. Lesions from the first institution were divided into a training cohort and a time-independent internal validation cohort with the ratio of 2:1. Lesions from the second institution were included as an external validation cohort. We evaluated the identification ability of each model by using the receiver operating characteristic (ROC) curve with area under curve (AUC). DeLong tests were used to evaluate the differences of predictive performance among models.

#### Radiomics Model Construction

##### Image segmentation

The region of interest (ROI) was manually delineated using ITK-SNAP software (version 2.2.0; www.itksnap.org) on the five sequences (T1W, T2W, AP, PVP, and ADC) images. We constructed two sets of ROIs. The first set of ROIs was delineated along the edge of each target tumor on each consecutive imaging slice to cover the whole tumoral volume. The second set of ROIs was restricted to the TLI zone, which was obtained by subtracting an inner segmentation from an outer segmentation. The outer segmentation was drawn ~2 mm outside the tumor boundary, and the inner segmentation was ~2 mm inside the tumor boundary.

##### Radiomic features extraction

Radiomic features were respectively extracted from the two sets of ROIs, including 18 first-order statistical features and 74 texture features. The first-order statistical features describe the distribution of voxel intensities in MR images. Textural features that describe the internal heterogeneity of ROI are calculated based on five textural matrices (Appendix E1). Additionally, the three-dimensional coiflets wavelet transformation was applied to decouple texture information for all patient data. Radiomic features were derived from the original image and eight wavelet decompositions for each patient both in TLI and tumor zone. The detailed illustration of these features is shown in Table E2.

##### Feature selection

The intra/interclass correlation coefficient of each feature was calculated to select the stable feature with the threshold of 0.8 (23) (Figures E1, E2). After pre-screening, we used the robust feature selection (RFS) method to select radiomic features. The RFS method emphasizes the minimization of joint *l*_2,1_-norm in loss function and regularization, and selects the features of joint sparsity on all data points (24). We ranked the coefficients of all features and top best features were selected as the effective predictors for later decision tree classifier.

##### Radiomics signature construction

The decision tree was applied to evaluate the ability of each of the five sequences for predicting HGP. We implemented this algorithm by tuning two parameters, the maximum sample of leaf and the maximum node, in the training process based on the selected features. We obtained five signatures after separately applying this algorithm on the five sequences both in the TLI and in the tumor zone. The forward stepwise regression method was used to select the desired sequences from five signatures, and the final radiomics signature was generated based on the desired sequences through logistics regression method. The decision tree algorithm was implemented by using the Python, version 3.6.5 “scikit-learn” package.

##### Cross validation

In order to elude the effect of a training/validation cohort split, we performed cross-validation between institutions. Lesions from the second institution were also divided into a training cohort and a time-independent internal validation cohort with the ratio of 2:1, and radiomics analysis was repeated in the final selected sequences. Lesions from the second institution were used as an external validation cohort.

#### Clinical and Combination Model Construction

Univariate analysis was used to evaluate the significance of clinical and qualitative imaging features in predicting HGPs of CRLMs. The forward stepwise was used to select desired features and construct the clinical model. In order to explore the complementarity between radiomics signature and clinical and qualitative imaging features, a combination model that incorporated the selected clinical and qualitative imaging features and radiomics signature was developed through logistic regression method. The forward stepwise was also used to selected the optimal factors for combination model.

### Nomogram Establishment

To offer a quantitative tool to investigate the ability of the combination model for HGP differentiation, we built a nomogram based on the multivariable logistic regression in the training cohort. Plotting calibration curves to evaluate the degree of deviation between the predictions and observed outcomes with Hosmer–Lemeshow test. Moreover, the decision curve analysis (DCA) was carried out to appraise its clinical usefulness by quantifying the net benefit under all threshold probabilities.

### Statistical Analysis

The differences of clinical factors between training and validation cohorts were verified by Student's t or Chi-square test. The quantitative variables were displayed by mean and standard deviation (SD). For categorical variables, number (*n*) and percentage (%) were used. These analyses were carried out on PASW Statistics version 25.0 (SPSS Inc., Chicago, IL, USA).

## Results

### Clinical and Histopathologic Characteristics

Based on the inclusion and exclusion criteria, a total of 107 patients were involved, including 43 women (40.2%) and 64 men (59.8%), with a median age of 59.79 years (interquartile range, 52–67 years). The median interval between MRI examination and hepatectomy was 17.02 days (interquartile range, 6.75–27.75 days).

Finally, a total of 195 CRLMs were recognized. The average number of lesions of each patient was 1.84 (range, 1–12); 38 patients had multiple CRLMs. There were 59 (30.3%) lesions with desmoplastic HGP (Figure E3a), 123 (63.1%) lesions with replacement HGP (Figure E3b), 10 lesions (5.1%) with pushing HGP, and three lesions (1.5%) with mixed HGP based on the international guidelines for scoring HGPs of liver metastases (3). Only one patient had both replacement and mixed CRLMs. Because of the low prevalence of pushing and mixed HGP, they were excluded from the study. This study finally analyzed 59 desmoplastic HGPs and 123 replacement HGPs.

The lesions from the first institution were grouped into training (*n* = 61) and internal validation (*n* = 31) cohort based on the surgery date. The external validation cohort was consisted of 90 lesions from the second institution. Baseline characteristics of these three cohorts are summarized in Table 1. No significant differences were observed for baseline clinical and qualitative imaging characteristics between the training and internal validation cohort (all *p* > 0.05). Compared with the training cohort, the external validation cohort showed significantly younger age and different occurrence time of the CRLMs; other characteristics were comparable between two cohorts.

**Table 1:** Demographics of patients.

**Characteristics**	**Training cohort (*n* = 61)**	**Internal validation cohort (*n* = 31)**	***p*-value**	**External validation cohort (*n* = 90)**	***p*-value**
Age [years, mean (SD)]	61.74 (9.66)	60.35 (10.42)	0.529	57.71 (10.05)	0.030^*^
Sex [*n* (%)]			0.804		
Male	39 (63.9)	19 (61.3)		53 (58.9)	0.533
Female	22 (36.1)	12 (38.7)		37 (41.1)	
Synchronous [*n* (%)]			0.572		0.001^*^
Yes	52 (85.2)	25 (80.6)		55 (61.1)	
No	9 (14.8)	6 (19.4)		35 (38.9)	
Primary site [*n* (%)]			0.834		0.395
Left-sided	44 (72.1)	23 (74.2)		59 (65.6)	
Right-sided	17 (27.9)	8 (25.8)		31 (34.4)	
Pathology [*n* (%)]			0.950		0.058
Low differentiation	35 (57.4)	18 (58.1)		65 (72.2)	
High and moderate differentiation	26 (42.6)	13 (41.9)		25 (27.8)	
Diameter [mm, mean (SD)]	26.69 (18.24)	33.17 (29.57)	0.198	24.00 (14.52)	0.382
Metastatic site [*n* (%)]			0.409		0.324
Segment I–IV	23 (37.7)	9 (29)		27 (30.0)	
Segment V–VIII	38 (62.3)	22 (71)		63 (70.0)	
Margin			0.312		0.055
Lobular	12 (19.7)	9 (29)		8 (8.9)	
Non-lobular	49 (80.3)	22 (71)		82 (91.1)	
Enhanced rim on AP			0.714		0.067
Yes	41 (67.2)	22 (71)		47 (52.2)	
No	20 (32.8)	9 (29)		43 (47.8)	
Enhanced rim on PVP			0.294		0.724
Yes	43 (70.5)	25 (80.6)		61 (67.8)	
No	18 (29.5)	6 (19.4)		29 (32.2)	
Pattern [*n* (%)]			0.959		0.942
Desmoplastic	20 (32.8)	10 (32.3)		29 (32.2)	
Replacement	41 (67.2)	21 (67.7)		61 (67.8)	

n, number of CRLMs; SD, standard deviation; AP, arterial phase; PVP, portal venous phase;

* *P < 0.05 showed significantly different*.

### Performance of the Clinical Model

**Figure 2:**
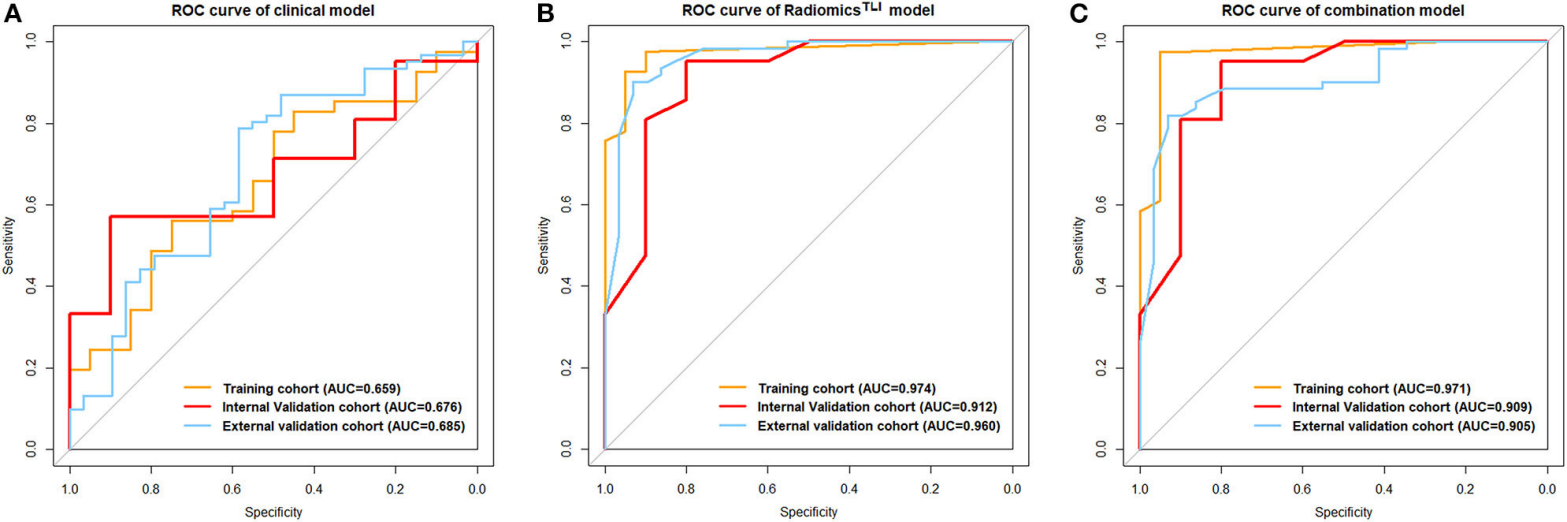
**(A)** ROC curve for the clinical model. **(B)** ROC curves for the radiomics^TLI^ model. **(C)** ROC curves for the combination model.

After univariate analysis, only lobular margin was statistically significant (*P* = 0.032, Table 2). The clinical model was constructed by sex, diameter, primary site, and lobular margin through forward stepwise regression. The AUC was 0.659 (95% CI: 0.514–0.803), 0.676 (95% CI: 0.484–0.869), and 0.685 (95% CI: 0.563–0.807) in three cohorts, respectively (Table 3). The ROC curves of the clinical model in all cohorts are displayed in Figure 2A.

**Table 2:** Univariate analysis of clinical and qualitative imaging features in distinguishing HGPs of CRLMs.

**Characteristics**	**Desmoplastic (*n* = 41)**	**Replacement (*n* = 20)**	**OR**	**95%CI**	***p*-value**
Sex (male)	28 (68.3%)	11 (55%)	1.84	(0.75–4.49)	0.182
Diameter	27.19 (19.74)	25.67 (15.11)	0.99	(0.97–1.00)	0.138
Metastatic site (segment I–IV)	16 (39.0%)	7 (35%)	1.73	(0.70–4.26)	0.233
Pathology (high/moderate differentiation)	22 (53.7%)	13 (65.0%)	1.42	(0.58–3.48)	0.440
Age	62.07 (9.03)	61.05 (11.06)	1.00	(0.96–1.05)	0.907
Synchronous	35 (85.4%)	17 (85.0%)	1.04	(0.32–3.37)	0.948
Primary site (left-sided colon)	32 (78.0%)	12 (60.0%)	0.50	(0.19–1.31)	0.158
Lobular margin	7 (17.1%)	5 (25.0%)	0.33	(0.13–0.91)	0.032^*^
Enhanced Rim on AP	26 (63.4%)	15 (75.0%)	0.90	(0.35–2.32)	0.827
Enhanced Rim on PP	30 (73.2%)	13 (65.0%)	1.34	(0.51–3.56)	0.553

n, number of CRLMs;

* *P < 0.05 showed statistical significance; OR, Odds ratio*.

**Table 3:** Performance of the clinical, radiomics, and combined models in distinguishing HGPs of CRLMs.

**Models**		**Clinical model**	**Radiomics^**tumor**^ model**	**Radiomics^**TLI**^ model**	**Combination model**
Training cohort	AUC	0.659 (0.514–0.803)	0.999 (0.997–1.000)	0.974 (0.940–1.000)	0.971 (0.927–1.000)
	ACC	0.623	0.983	0.934	0.967
	SEN	0.561	0.976	0.950	0.976
	SPE	0.750	1.000	0.927	0.950
Internal validation cohort	AUC	0.676 (0.484–0.869)	0.879 (0.741–1.000)	0.912 (0.789–1.000)	0.909 (0.785–1.000)
	ACC	0.613	0.774	0.903	0.871
	SEN	0.571	0.762	0.952	0.952
	SPE	0.700	0.800	0.800	0.700
External validation cohort	AUC	0.685 (0.563–0.807)	—	0.960 (0.919–1.000)	0.905 (0.841–0.970)
	ACC	0.567	—	0.811	0.788
	SEN	0.475	—	1.000	1.000
	SPE	0.759	—	0.414	0.345

*AUC, area under curve; ACC, accuracy; SEN, sensitivity; SPE, specificity*.

### Performance of Tree-Based Radiomics Signatures

We extracted 828 features for each sequence. After inter/intra-observer agreement analysis, the dimensional of feature space was 679 for T2WI, 483 for ADC, 631 for T1WI, 737 for AP, and 691 for PVP images in TLI zone; and 652 for T2WI, 549 for ADC, 594 for T1WI, 694 for AP, and 679 for PVP images in tumor zone (Figures E1, E2). The RFS method ranked radiomic features and the top 20 most discriminative features were used to construct the decision tree classifier (25). The details of these selected features for each sequence are demonstrated in Tables E3–E12.

When comparing the ability for HGP differentiation of each single sequence in internal validation cohort, signature^AP^ derived from the ROI of TLI zone exhibited the best performance in all sequences, and signature^T1WI^ derived from the ROI of tumor zone performed the best in all sequences (Table 4). The final radiomics^TLI^ signature was generated by signature^T2WI^, signature^AP^, and signature^PVP^ in TLI zone through forward stepwise regression (Table E13), and the radiomics^tumor^ signature was generated by signature^T1WI^, signature^T2WI^, signature^AP^, and signature^PVP^ in tumor zone in the same way. The radiomics^TLI^ signature demonstrated better performance than the radiomics^tumor^ signature in the internal validation cohort (AUC: 0.912 [95% CI: 0.789–1] vs. 0.879 [95% CI: 0.741–1], Table 3 and Figure 2B). The Delong test showed that the performance of the radiomics^TLI^ model was obviously better than that of the clinical model (*P* = 0.035), while the radiomics^tumor^ model did not show significant better performance than the clinical model in the internal validation cohort (*P* = 0.051, Table 5).

**Table 4:** Performance of each single sequence in distinguishing HGPs of CRLMs.

**Model**		**Training cohort**				**Internal validation cohort**			
		**AUC**	**ACC**	**SEN**	**SPE**	**AUC**	**ACC**	**SEN**	**SPE**
TLI area	T1WI	0.707 (0.599–0.816)	0.803	1.000	0.400	0.619 (0.458–0.780)	0.710	0.905	0.300
	T2WI	0.845 (0.769–0.921)	0.803	0.854	0.700	0.767 (0.592–0.942)	0.677	0.905	0.200
	AP	0.907 (0.832–0.982)	0.885	0.878	0.900	0.860 (0.721–0.999)	0.806	0.905	0.600
	PVP	0.812 (0.712–0.912)	0.803	0.707	1.000	0.736 (0.590–0.882)	0.677	0.571	0.900
	ADC	0.754 (0.645–0.863)	0.721	0.659	0.850	0.733 (0.567–0.900)	0.710	0.667	0.800
Tumor area	T1WI	0.902 (0.825–0.980)	0.836	0.805	0.900	0.843 (0.708–0.978)	0.806	0.857	0.700
	T2WI	0.762 (0.640–0.884)	0.754	0.780	0.700	0.657 (0.469–0.845)	0.677	0.714	0.600
	AP	0.840 (0.725–0.954)	0.852	0.902	0.750	0.679 (0.493–0.864)	0.677	0.667	0.700
	PVP	0.887 (0.798–0.975)	0.869	0.902	0.800	0.717 (0.533–0.900)	0.710	0.714	0.700
	ADC	0.916 (0.848–0.985)	0.902	1.000	0.700	0.833 (0.713–0.954)	0.839	1.000	0.500

**Table 5:** Delong tests' results between different models.

**Cohorts**	**Clinical and radiomics^**TLI**^**	**Clinical and combination**	**Radiomics^**TLI**^ and combination**	**Clinical and radiomics^**tumor**^**	**Radiomics^**TLI**^ and Radiomics^**tumor**^**
Training	<0.001^*^	<0.001^*^	0.719	<0.001^*^	0.152
Validation	0.035^*^	0.034^*^	0.916	0.051	0.607

* *P-value < 0.05 showed statistical significance*.

### Performance of Combination Model and Nomogram

**Figure 3:**
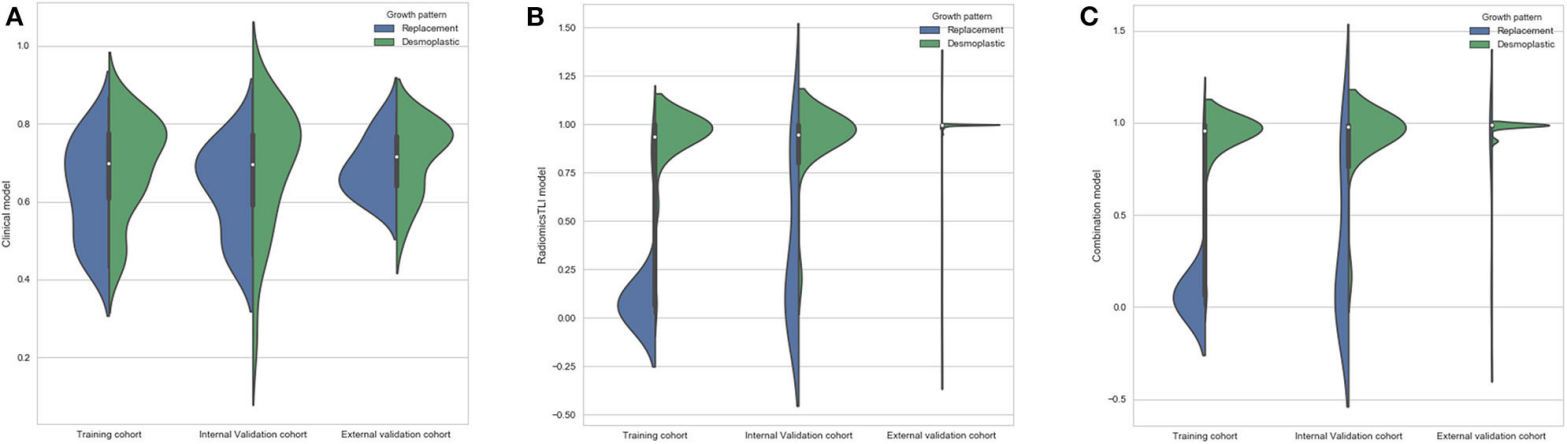
**(A)** Violin graph of distribution of clinical model between replacement and desmoplastic HGPs. **(B)** Violin graph of distribution of radiomics^TLI^ model between replacement and desmoplastic HGPs. **(C)** Violin graph of distribution of combination model between replacement and desmoplastic HGPs.

**Figure 4:**
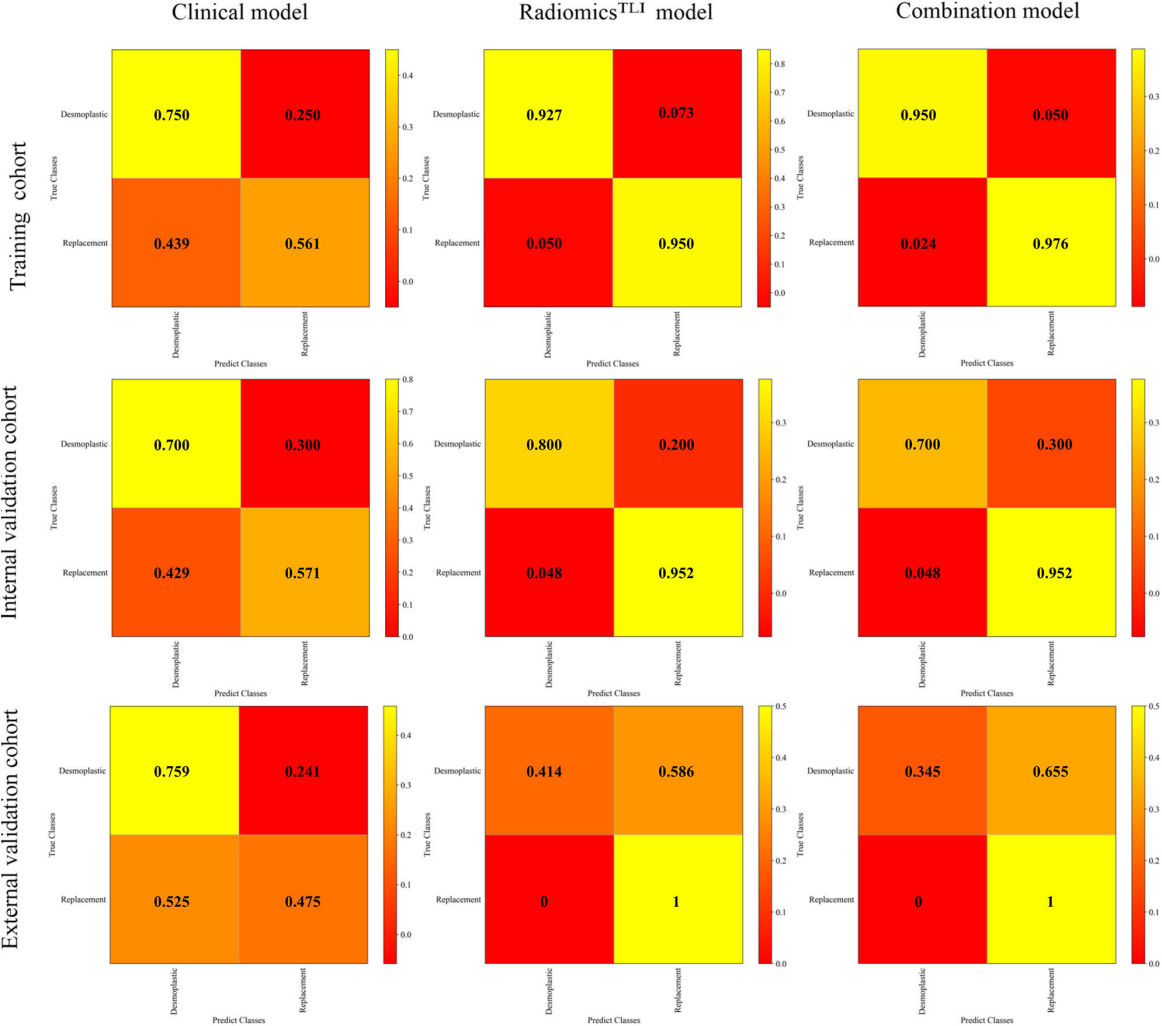
Confusion matrixes for clinical, radiomics^TLI^, and combination model in all cohorts.

The radiomics^TLI^ signature and clinical features were used to construct the combination model. The decision trees of TLI zone in T2WI, AP, and PVP images were shown in Figure E4. The formula of radiomics^TLI^ signature was exhibited in Appendix E2. The AUCs of combination model were 0.971, 0.909, and 0.905 in three cohorts, respectively (Table 3 and Figure 2C). According to the violin graphs (Figure 3), the distinguishing ability of combination model was much better than that of the clinical model (*P* = 0.034, Table 5), while there was no difference between the radiomics^TLI^ model and combination model (*P* = 0.916, Table 5). With respect to the predictive ability of each model in three cohorts, the confusion matrixes shown the sensibility, specificity, false positive and false negative by heat maps (Figure 4).

**Figure 5:**
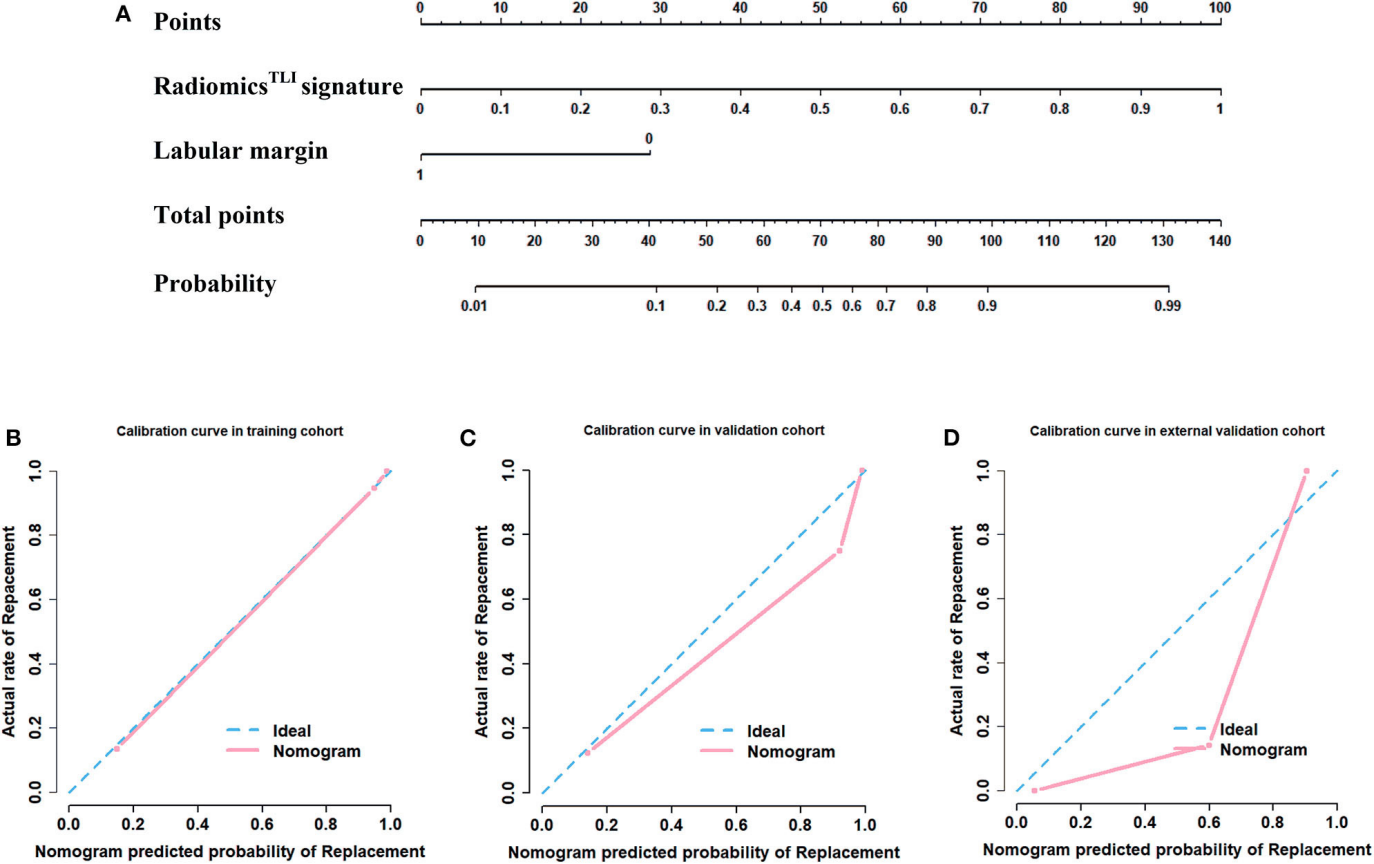
Development of nomogram and calibration curves. **(A)** Nomogram based on radiomics signatures and clinical factors. Calibration curves of the radiomics nomogram in the **(B)** training cohort, **(C)** internal validation cohort, and **(D)** external validation cohorts.

**Figure 6:**
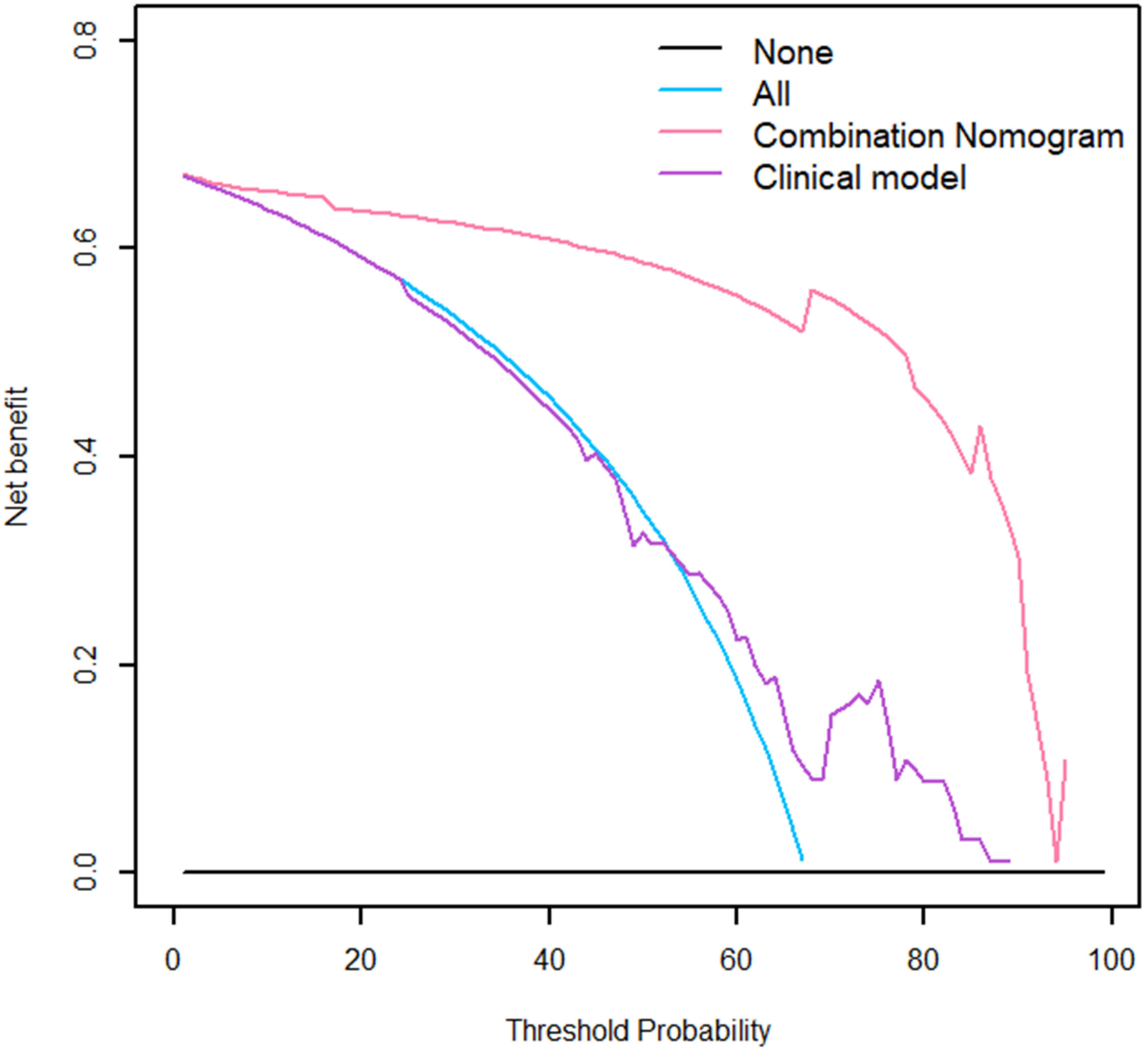
The decision curve of the nomogram.

The nomogram is showed in Figure 5. The AUC of combination nomogram was 0.971, 0.909, and 0.905 in three cohorts, respectively. The calibration curves for three cohorts were shown in Figures 5B–D. The Hosmer–Lemeshow test showed the predicted HGP was in consistency with the actual HGP (*P*-value: training cohort, 0.874; internal validation cohort, 0.346; external validation cohort, 0.101). The decision curves showed that the combination nomogram added more benefit than clinical nomogram if the threshold probability >3% (Figure 6).

### Performance of Cross Validation

The results of cross validation are shown in Table E14. The final radiomics^TLI^ model also shown good performance with AUC of 0.888 in the training cohort, 0.906 in the internal validation cohort, and 0.788 in the external validation cohort. The decision trees that was constructed from second institution was shown in Figure E5. We noticed some radiomic features were selected in both institutions and showed them in Table E15.

## Discussion

In this study, we demonstrated that a multiparameter MRI-based radiomics model could be used as a non-invasion tool to preoperatively distinguish the HGP of CRLMs. Radiomics analysis was performed on both TLI and tumor zones, and the radiomics^TLI^ model showed better performance than that of the radiomics^tumor^ model. The nomogram integrating the radiomics^TLI^ signature and clinical factors showed satisfactory performance in all cohorts.

Based on the discrepancy of microvasculature between desmoplastic (angiogenesis) and replacement (non-angiogenesis, but vessel co-option) HGPs, previous studies only focused on the degree of tumor angiogenesis (26) and the response to anti-angiogeneic treatment by using textures analyses (26–28). Most studies suggest that the higher score reflecting textural heterogeneity corresponds to higher degree of angiogenesis (24), which may be associated with more aggressiveness (29, 30). In contrast to these results, Ravanelli found that the CRLMs with more uniform textual features on contrast enhanced CT images had a worse objective response rates and shorter long-term survivals after treatment of Bevacizumab (29). However, Ravanelli didn't reveal the association between those textual features and pathological characteristics, such as the degree of angiogenesis or even HGPs.

Unlike previous studies, we created a multiparameter MRI-based radiomics model to predict HGPs with high accuracy. With pathology as reference, the desmoplastic HGP was identified with more heterogeneous radiomic features and the replacement ones had more homogeneous radiomics features (Tables E3–E12). Four radiomic features were selected in both institution after cross validation, including wavelet.LLH_glcm_Idmn in T2WI, wavelet.HHL_glcm_Correlation, and wavelet.HLH_firstorder_Skewness in AP images, and wavelet.HHL_glcm_InverseVariance in PVP images (Table E15). According the description of these features, the uniformity, symmetry, and variation of gray value are of great significance for HGP identification. The super enhanced small areas highlighted by image filtering and quantized by the variable uniformity may be caused by the leakage of contrast agent into the extracellular space around the highly permeable and newly formed tumor microvessels (29). Apart from the angiogeneic situation, there are more tissue types in the desmoplastic HGP tumor, such as fibrosis, inflammatory, tumor and liver cells, which shows higher heterogeneity than the replacement ones. Therefore, radiomic features of desmoplastic or replacement HGPs may be used as biomarker to predict response to bevacizumab and long-term prognosis.

Compared with the radiomic features, this study showed the gross features on MRI could not distinguish HGP effectively. It suggests that the traditional image analysis may over-simplify the tumor biology (30), including the growth pattern and corresponding microvasculature. Our previous study revealed that the enhanced rim on PVP CT images was correlated with the histopathological presentation of the desmoplastic HGP (19). Furthermore, a similar feature of transit enhanced rim on AP MR images was also associated with the desmoplastic reaction (11). However, this study showed no significant difference between enhanced rim and HGP. Compared with CT, the superior soft tissue resolution of MR may cause more common enhanced rim in MRI, which may explain enhanced rim is not selected in our study. Furthermore, considering the very limited cases in Semelka's study, more profound study may be required. The lobular margin was identified as a significant feature of replacement HGP through univariate analysis in our study. It was consistent with another study, in which lobular margin was identified as a poor prognostic predictor in patient with resected CRLMs (22). It is an important observation and may reflect the irregular border on HE-stained tissue sections in the replacement growth pattern.

According to the results, we learned both the radiomics^tumor^ and radiomics^TLI^ models were superior to the clinical model in all cohorts. Additionally, the Delong test showed no significant difference (*P* = 0.607) between the radiomics^tumor^ and radiomics^TLI^ model. It suggested that radiomics analysis in each ROI could be used to identify HGP of CRLMs independently. The radiomics^TLI^ model had a higher AUC than the radiomics^tumor^ model in the internal validation cohort, which could be due to the histological differences of HGPs are mainly focused on the perilesional zone, the features extracted from the whole tumor area might not reflect the differences in HGPs significantly. Therefore, the final combination model was constructed by radiomics^TLI^ signature and related clinical characteristics. We noticed that the performance of combination model was significantly better than that of clinical model (*P* < 0.05 in all cohorts), which indicated that the radiomics^TLI^ signature can complement the clinical characteristics. In addition, in external validation cohort, the sensitivity was higher and lesions were more likely to be predicted as a replacement HGP. However, due to the large proportion of replacement HGP, this may lead to good AUC, but poor calibration. The decision curve analysis demonstrated that the combination nomogram was superior to clinical nomogram, which enabled the evaluation of clinical relevance and verified the radiomics signature hold great potential for clinical application.

This study has some limitations. Firstly, the sample size of this study was small and it was a retrospective study. Sufficient data is the basis for performance evaluation of the predictive model in radiomics or elsewhere. Most patients with CRLMs are initially unsuitable for resection, which limits the chemo-naïve specimens. In addition, Gillies et al. suggested that although larger data sets provided more power, radiomics could be performed with as few as 100 samples (31). Although we used the external validation to reduce the impact, the prospective multi-center study was still required in the future and the survival prediction with this model could be tested. Secondly, the pushing and mixed HGPs were not evaluated because of the limited number. However, it was still consistent with the data published in the international consensus (3). Thirdly, few clinical factors were included. Considering the need for more data in machine learning, other important factors, such as disease-free interval, preoperative CEA, and genetic (BRAF/KRAS), were not included in this study. More samples and more clinical factors are essential to obtain a comprehensive model and higher accuracy in future. Finally, the radiomics model could be used to distinguish the predominant (>50% area) HGP of resectable CRLMs. Considering the inter- and intra-tumor heterogeneity of HGP types, it remains challenging whether and how the model can used in patients with unresectable CRLMs.

## Conclusion

In conclusion, MRI-based radiomic approach has high potential to predict the predominant HGPs of CRLM, paving the way for further validation in larger and possibly prospective datasets. Moreover, the results suggested that MRI can not only be used as an independent prediction tool for HGP but also improve the performance of clinical factors.

## Data Availability Statement

The datasets generated for this study are available on request to the corresponding author.

## Ethics Statement

The studies involving human participants were reviewed and approved by Medical Ethics Committee of Peking University People's Hospital. Written informed consent for participation was not required for this study in accordance with the national legislation and the institutional requirements.

## Author Contributions

YW contributed to the study concept and design. TT, WS, and NH contributed to the acquisition of clinical data. DG contributed to the data review. YH contributed to the data analysis and figures. FC, YYu, and YZ contributed to the data interpretation. FC wrote the first draft of the manuscript. JC and YH wrote the sections of the manuscript. YW, JW, and JT supervised and oversaw the study. All authors contributed to the manuscript revision, read, and approved the submitted version.

## Conflict of Interest

The authors declare that the research was conducted in the absence of any commercial or financial relationships that could be construed as a potential conflict of interest.
