# Supplementary material for: Identification of Predominant Histopathological Growth Patterns of Colorectal Liver Metastasis by Multi-Habitat and Multi-Sequence Based Radiomics Analysis
Source: Front Oncol. 2020 Aug 14;10:1363. doi: 10.3389/fonc.2020.01363 (PMC7456817; doi:10.3389/fonc.2020.01363)
Supplement: Supplementary file 1 [file Data_Sheet_1.docx]

Supplementary Material

# Appendix E1：Definitions of radiomic features used in this study.

Radiomic feature extraction of this study was based on Pyradiomics, including three types: non-textural, textural, and wavelet features. Non-textural features included the shape features (n=13) and first order statistical features (n=18). Textural features (n=74) were extracted based on five textural matrices: the Gray Level Co-occurrence Matrix (GLCM), the Gray Level Run-Length Matrix (GLRLM), the Gray Level Size Zone Matrix (GLSZM), the Neighborhood Gray-Tone Difference Matrix (NGTDM) and gray level dependence matrix (GLDM). The 3-dimensional wavelet transformation was applied to decompose the original image into 8 parts. The wavelet decompositions of original image were labeled as Wavelet.LLL, Wavelet.LLH, Wavelet.LHL, Wavelet.LHH, Wavelet.HLL, Wavelet.HLH, Wavelet.HLH, and Wavelet.HHH, where “L” and “H” represented low- and high-pass filters. For example, Wavelet.LLH represented the image filtered with low-pass in x- and y-directions and high-pass in z-directions, as follows:

$Wavelet.LLH(i,j,k)$= $\sum_{p=1}^{Nl} \sum_{q=1}^{Nl} \sum_{r=1}^{Nh} L(p)L(q)H(r)X(i+p, j+q,k+r)$,

where $Nl$ is the length of filter L, Nh is the length of filter H and X is the original image.

Types and names of the features are shown in Table E1. We finally extracted 841 radiomic features, including shape-based feature in original image, first order features and textural features in original and wavelet images (18*9+74*9 = 828). All the feature extraction was implemented using Python 3.6.5.

# Appendix E2：The formula of radiomics^TLI^ signature.

The final radiomics^TLI^ signature was generated by signature^T2WI^, signature^AP^ and signature^PVP^ in TLI zone as follow:

$$Radiomics signature= \frac{exp(-3.927+1.268*{signature}^{T2WI}+4.327*{signature}^{AP}+6.277*{signature}^{PVP})}{1+exp(-3.927+1.268*{signature}^{T2WI}+4.327*{signature}^{AP}+6.277*{signature}^{PVP})}$$

# Appendix E3：Supplementary Tables and Figures.

## Supplementary Tables

**Table E1. Sequence parameters for multiparametric MRI**

| **Parameters** | **T2W** | **T1W** | **AP** | **PVP** | **DWI** |
| --- | --- | --- | --- | --- | --- |
| **TR/TE(ms)** | 5000/96 | 5.116/1.674 | 5.116/1.674 | 5.116/1.674 | 6000/58 |
| **Field of view(FOV, cm)** | 38 | 36 | 36 | 36 | 38 |
| **Matrix size** | 320×320 | 280×170 | 280×170 | 280×170 | 160×160 |
| **Thickness(mm)** | 7 | 5 | 5 | 5 | 7 |
| **Gap(mm)** | 1 | 2.5 | 2.5 | 2.5 | 1 |
| **Other** |  |  |  |  | b values= 0, 800 mm^2^/sec |

Note: The contrast administration consisted of 0.25mmol/kg gadopentetate dimeglumine at a flow rate of 2.5 mL/s, followed by a flush of 20 mL of saline (2 mL/sec) with a power injector (Spectris Solaris Injection System; Bayer HealthCare Pharmaceuticals, Berlin, Germany).

**Table E2. Radiomic features used in this study.**

| **Non-textural feature (n=18)** |
| --- |
| 10th Percentile |
| 90th Percentile |
| Energy |
| Entropy |
| Interquartile Range |
| Kurtosis |
| Maximum |
| Mean |
| Mean Absolute Deviation |
| Median |
| Minimum |
| Range |
| Robust Mean Absolute Deviation |
| Root Mean Squared |
| Skewness |
| Total Energy |
| Uniformity |
| Variance |
| **Texture feature (n =74)** |
| **GLCM (n=23)** |
| Autocorrelation |
| Cluster Prominence |
| Cluster Shade |
| Cluster Tendency |
| Contrast |
| Correlation |
| Difference Average |
| Difference Entropy |
| Difference Variance |
| Inverse Difference (Id) |
| Inverse Difference Moment (Idm) |
| Inverse Difference Moment Normalized (Idmn) |
| Inverse Difference Normalized (Idn) |
| Informational Measure of Correlation 1 (Imc1) |
| Informational Measure of Correlation 2 (Imc2) |
| Inverse Variance |
| Joint Average |
| Joint Energy |
| Joint Entropy |
| Maximum Probability |
| Sum Average |
| Sum Entropy |
| Sum Squares |
| **GLDM (n=14)** |
| Dependence Entropy |
| Dependence Non-Uniformity |
| Dependence Non-Uniformity Normalized |
| Dependence Variance |
| Gray Level Non-Uniformity |
| Gray Level Variance |
| High Gray Level Emphasis |
| Large Dependence Emphasis |
| Large Dependence High Gray Level Emphasis |
| Large Dependence Low Gray Level Emphasis |
| Low Gray Level Emphasis |
| Small Dependence Emphasis |
| Small Dependence High Gray Level Emphasis |
| Small Dependence Low Gray Level Emphasis |
| **GLRLM (n=16)** |
| Gray Level Non-Uniformity |
| Gray Level Non-Uniformity Normalized |
| Gray Level Variance |
| High Gray Level Run Emphasis |
| Long Run Emphasis |
| Long Run High Gray Level Emphasis |
| Long Run Low Gray Level Emphasis |
| Low Gray Level Run Emphasis |
| Run Entropy |
| Run Length Non-Uniformity |
| Run Length Non-Uniformity Normalized |
| Run Percentage |
| Run Variance |
| Short Run Emphasis |
| Short Run High Gray Level Emphasis |
| Short Run Low Gray Level Emphasis |
| **GLSZM (n=16)** |
| Gray Level Non-Uniformity |
| Gray Level Non-Uniformity Normalized |
| Gray Level Variance |
| High Gray Level Zone Emphasis |
| Large Area Emphasis |
| Large Area High Gray Level Emphasis |
| Large Area Low Gray Level Emphasis |
| Low Gray Level Zone Emphasis |
| Size Zone Non-Uniformity |
| Size Zone Non-Uniformity Normalized |
| Small Area Emphasis |
| Small Area High Gray Level Emphasis |
| Small Area Low Gray Level Emphasis |
| Zone Entropy |
| Zone Percentage |
| Zone Variance |
| **NGTDM (n=5)** |
| Busyness |
| Coarseness |
| Complexity |
| Contrast |
| Strength |

**Table E3. The averages and standard deviations of the top** **20 features in desmoplastic and replacement HGP CRLMs used in TLI area of T1W images**

| NO. | Feature | Desmoplastic | Replacement | p |
| --- | --- | --- | --- | --- |
| 1 | wavelet.HHH_glcm_Idm | 0.767(0.013) | 0.758(0.015) | 0.014 |
| 2 | wavelet.HHH_glcm_Id | 0.767(0.013) | 0.759(0.014) | 0.017 |
| 3 | wavelet.LLH_glszm_SizeZoneNonUniformityNormalized | 0.146(0.043) | 0.180(0.044) | 0.005 |
| 4 | wavelet.HHH_glcm_DifferenceAverage | 0.466(0.027) | 0.488(0.035) | 0.009 |
| 5 | wavelet.HHH_glcm_Correlation | 0.072(0.050) | 0.045(0.049) | 0.048 |
| 6 | wavelet.HHH_glszm_ZonePercentage | 0.003(0.003) | 0.007(0.007) | 0.005 |
| 7 | wavelet.HHH_glcm_InverseVariance | 0.463(0.025) | 0.476(0.025) | 0.070 |
| 8 | wavelet.HHH_firstorder_10Percentile | -2.454(2.475) | -3.966(2.994) | 0.041 |
| 9 | wavelet.LHH_glcm_Idm | 0.796(0.0.025) | 0.781(0.032) | 0.030 |
| 10 | wavelet.LHH_glcm_Id | 0.798(0.024) | 0.783(0.029) | 0.030 |
| 11 | wavelet.HLH_glcm_Correlation | 0.166(0.059) | 0.124(0.069) | 0.018 |
| 12 | wavelet.HHL_glszm_GrayLevelNonUniformity | 15.248(23.647) | 38.578(60.562) | 0.034 |
| 13 | wavelet.HHH_firstorder_RootMeanSquared | 2.622(2.175) | 4.179(2.986) | 0.024 |
| 14 | wavelet.HHH_glrlm_GrayLevelNonUniformityNormalized | 0.498(0.005) | 0.491(0.018) | 0.025 |
| 15 | wavelet.LHH_gldm_SmallDependenceEmphasis | 0.016(0.007) | 0.021(0.010) | 0.048 |
| 16 | wavelet.LHH_glcm_DifferenceAverage | 0.408(0.056) | 0.447(0.077) | 0.031 |
| 17 | wavelet.LHH_glrlm_GrayLevelNonUniformityNormalized | 0.489(0.024) | 0.477(0.040) | 0.150 |
| 18 | wavelet.HHH_firstorder_Uniformity | 0.499(0.003) | 0.494(0.012) | 0.031 |
| 19 | wavelet.LHH_firstorder_Uniformity | 0.493(0.017) | 0.485(0.029) | 0.167 |
| 20 | wavelet.HHH_glcm_JointEnergy | 0.254(0.004) | 0.249(0.011) | 0.024 |

**Table E4. The averages and standard deviations of the top 20 features in desmoplastic and replacement HGP CRLMs used in TLI area of T2W images**

| NO. | Feature | Desmoplastic | Replacement | p |
| --- | --- | --- | --- | --- |
| 1 | wavelet.LHL_glcm_InverseVariance | 0.406(0.067) | 0.392(0.084) | 0.511 |
| 2 | wavelet.HHH_glcm_InverseVariance | 0.498(0.029) | 0.476(0.058) | 0.047 |
| 3 | wavelet.HHH_firstorder_Kurtosis | 7.771(4.314) | 5.680(2.349) | 0.048 |
| 4 | wavelet.HLL_glcm_InverseVariance | 0.411(0.059) | 0.382(0.084) | 0.121 |
| 5 | wavelet.HLH_glcm_InverseVariance | 0.425(0.076) | 0.386(0.078) | 0.066 |
| 6 | original_firstorder_Median | 67.524(38.298) | 110.338(101.319) | 0.020 |
| 7 | wavelet.LHH_firstorder_Kurtosis | 6.233(3.702) | 4.644(1.726) | 0.074 |
| 8 | original_firstorder_10Percentile | 49.843(29.178) | 84.871(85.014) | 0.020 |
| 9 | wavelet.LLL_firstorder_Median | 192.559(107.843) | 312.839(287.789) | 0.021 |
| 10 | wavelet.HHH_firstorder_Skewness | 0.051(0.280) | -0.090(0.252) | 0.062 |
| 11 | wavelet.LLL_firstorder_Mean | 199.220(114.070) | 317.071(290.446) | 0.026 |
| 12 | wavelet.LLL_firstorder_RootMeanSquared | 204.672(117.656) | 323.677(294.459) | 0.028 |
| 13 | wavelet.LLH_glcm_Idmn | 0.962(0.032) | 0.974(0.016) | 0.119 |
| 14 | wavelet.LLL_firstorder_10Percentile | 146.111(85.297) | 242.073(242.573) | 0.026 |
| 15 | original_firstorder_Mean | 72.135(42.669) | 113.230(103.785) | 0.032 |
| 16 | wavelet.LLH_firstorder_Median | 4.992(12.250) | -5.907(15.863) | 0.004 |
| 17 | original_firstorder_RootMeanSquared | 75.251(45.346) | 116.168(105.643) | 0.038 |
| 18 | wavelet.LLL_firstorder_90Percentile | 263.771(160.132) | 397.098(348.944) | 0.044 |
| 19 | wavelet.LLH_glrlm_RunLengthNonUniformityNormalized | 0.903(0.079) | 0.861(0.131) | 0.123 |
| 20 | wavelet.LLL_firstorder_Minimum | 101.703(72.078) | 168.267(198.852) | 0.060 |

**Table E5.** **The averages and standard deviations of the top** **20 features in desmoplastic and replacement HGP CRLMs used in TLI area of arterial phase images**

| NO. | Feature | Desmoplastic | Replacement | p |
| --- | --- | --- | --- | --- |
| 1 | wavelet.HLH_firstorder_Skewness | 0.141(0.368) | -0.090(0.260) | 0.015 |
| 2 | wavelet.HHL_glcm_Correlation | 0.173(0.061) | 0.135(0.066) | 0.036 |
| 3 | wavelet.LHH_gldm_DependenceNonUniformityNormalized | 0.065(0.009) | 0.072(0.013) | 0.009 |
| 4 | wavelet.LHH_gldm_DependenceVariance | 19.251(3.783) | 16.455(4.256) | 0.012 |
| 5 | wavelet.HHH_glcm_InverseVariance | 0.486(0.019) | 0.497(0.015) | 0.022 |
| 6 | wavelet.HHH_glcm_Id | 0.750(0.015) | 0.729(0.034) | 0.001 |
| 7 | wavelet.HHH_glcm_Idm | 0.749(0.017) | 0.724(0.042) | 0.002 |
| 8 | wavelet.HHH_glcm_Correlation | 0.028(0.035) | 0.005(0.029) | 0.016 |
| 9 | wavelet.HHH_firstorder_InterquartileRange | 5.572(3.880) | 9.540(5.535) | 0.002 |
| 10 | wavelet.HHL_glcm_InverseVariance | 0.431(0.038) | 0.459(0.027) | 0.005 |
| 11 | wavelet.HLH_glcm_InverseVariance | 0.447(0.031) | 0.465(0.023) | 0.019 |
| 12 | wavelet.HLH_gldm_DependenceNonUniformityNormalized | 0.067(0.009) | 0.074(0.013) | 0.013 |
| 13 | wavelet.HHH_firstorder_RobustMeanAbsoluteDeviation | 2.461(1.577) | 4.113(2.344) | 0.002 |
| 14 | wavelet.HHL_gldm_DependenceVariance | 18.898(4.766) | 15.878(4.591) | 0.022 |
| 15 | wavelet.HHH_glrlm_ShortRunEmphasis | 0.702(0.031) | 0.733(0.041) | 0.002 |
| 16 | wavelet.HHH_glcm_DifferenceAverage | 0.508(0.040) | 0.575(0.120) | 0.002 |
| 17 | wavelet.HHH_firstorder_Uniformity | 0.488(0.017) | 0.465(0.049) | 0.007 |
| 18 | wavelet.HHH_glrlm_RunPercentage | 0.643(0.036) | 0.676(0.045) | 0.004 |
| 19 | wavelet.HHH_firstorder_10Percentile | -7.015(3.863) | -10.958(6.126) | 0.003 |
| 20 | wavelet.LHL_glcm_InverseVariance | 0.395(0.068) | 0.369(0.096) | 0.221 |

**Table E6. The averages and standard deviations of the top 20 features in desmoplastic and replacement HGP CRLMs used in TLI area of portal venous phase image**

| NO. | Feature | Desmoplastic | Replacement | p |
| --- | --- | --- | --- | --- |
| 1 | wavelet.HHL_glcm_InverseVariance | 0.416(0.043) | 0.462(0.027) | <0.001 |
| 2 | wavelet.HHH_glcm_Correlation | 0.045(0.053) | 0.008(0.026) | 0.005 |
| 3 | wavelet.HHL_gldm_LargeDependenceEmphasis | 138.376(52.420) | 89.102(35.996) | <0.001 |
| 4 | wavelet.HHL_glrlm_LongRunEmphasis | 3.875(1.225) | 2.696(0.728) | <0.001 |
| 5 | wavelet.HHL_glrlm_RunVariance | 1.034(0.447) | 0.618(0.248) | <0.001 |
| 6 | wavelet.HHL_glcm_Correlation | 0.192(0.076) | 0.142(0.069) | 0.014 |
| 7 | wavelet.HHL_glrlm_ShortRunEmphasis | 0.686(0.095) | 0.773(0.071) | 0.001 |
| 8 | wavelet.HHH_glrlm_ShortRunEmphasis | 0.694(0.043) | 0.734(0.040) | 0.001 |
| 9 | wavelet.HHH_glrlm_LongRunEmphasis | 3.527(0.605) | 2.984(0.478) | 0.001 |
| 10 | wavelet.HHL_glrlm_RunPercentage | 0.631(0.100) | 0.718(0.074) | 0.001 |
| 11 | wavelet.HHH_gldm_LargeDependenceEmphasis | 123.649(25.174) | 101.229(22.340) | 0.001 |
| 12 | wavelet.HHH_glcm_InverseVariance | 0.478(0.028) | 0.495(0.013) | 0.014 |
| 13 | wavelet.HHH_firstorder_10Percentile | -5.974(4.798) | -10.304(5.184) | 0.002 |
| 14 | wavelet.HHH_glcm_Id | 0.754(0.022) | 0.734(0.027) | 0.002 |
| 15 | wavelet.HHL_glrlm_RunLengthNonUniformityNormalized | 0.470(0.121) | 0.571(0.095) | 0.002 |
| 16 | wavelet.HHL_glcm_JointEnergy | 0.204(0.076) | 0.141(0.063) | 0.002 |
| 17 | wavelet.HHH_glcm_Idm | 0.753(0.024) | 0.729(0.033) | 0.002 |
| 18 | wavelet.HHH_glrlm_RunPercentage | 0.637(0.046) | 0.678(0.045) | 0.001 |
| 19 | wavelet.HHH_firstorder_90Percentile | 5.906(4.673) | 10.140(5.107) | 0.002 |
| 20 | wavelet.HHL_glrlm_GrayLevelNonUniformityNormalized | 0.411(0.101) | 0.332(0.089) | 0.004 |

**Table E7. The averages and standard deviations of the top 20 features in desmoplastic and replacement HGP CRLMs used in TLI area of ADC image**

| NO. | Feature | Desmoplastic | Replacement | p |
| --- | --- | --- | --- | --- |
| 1 | wavelet.HLL_ngtdm_Complexity | 0.382(0.037) | 0.344(0.060) | 0.003 |
| 2 | wavelet.HLL_ngtdm_Contrast | 0.094(0.010) | 0.085(0.016) | 0.006 |
| 3 | wavelet.LLH_glrlm_ShortRunHighGrayLevelEmphasis | 1.676(0.392) | 1.628(0.378) | 0.647 |
| 4 | wavelet.LLL_glcm_Imc1 | -0.011(0.036) | -0.367(0.075) | 0.079 |
| 5 | wavelet.LHH_glcm_InverseVariance | 0.487(0.053) | 0.472(0.079) | 0.375 |
| 6 | wavelet.LHH_glcm_DifferenceAverage | 0.487(0.053) | 0.472(0.079) | 0.375 |
| 7 | wavelet.LHH_glcm_Contrast | 0.487(0.053) | 0.472(0.079) | 0.375 |
| 8 | wavelet.LHH_glszm_ZoneEntropy | 2.514(0.794) | 2.643(0.820) | 0.559 |
| 9 | wavelet.LHL_firstorder_InterquartileRange | 0.0002(<0.001) | 0.0001(<0.001) | 0.006 |
| 10 | wavelet.LLH_firstorder_10Percentile | -0.001(<0.001) | -0.001(0.001) | 0.167 |
| 11 | wavelet.LHL_firstorder_RobustMeanAbsoluteDeviation | 0.0001(<0.001) | 0.0001(<0.001) | 0.007 |
| 12 | wavelet.LLH_ngtdm_Complexity | 0.325(0.104) | 0.301(0.131) | 0.443 |
| 13 | wavelet.LHL_firstorder_90Percentile | 0.0002(<0.001) | 0.0001(<0.001) | 0.005 |
| 14 | original_gldm_LargeDependenceLowGrayLevelEmphasis | 125.288(84.908) | 122.476(109.074) | 0.912 |
| 15 | wavelet.HLL_glrlm_ShortRunLowGrayLevelEmphasis | 0.430(0.053) | 0.420(0.062) | 0.542 |
| 16 | wavelet.LLL_firstorder_Maximum | 0.006(0.002) | 0.007(0.002) | 0.030 |
| 17 | wavelet.LLH_glrlm_HighGrayLevelRunEmphasis | 2.624(0.247) | 2.444(0.423) | 0.040 |
| 18 | original_glcm_Imc1 | -0.024(0.048) | -0.045(0.074) | 0.190 |
| 19 | original_glcm_Imc2 | 0.030(0.070) | 0.060(0.100) | 0.173 |
| 20 | wavelet.LLH_glrlm_LowGrayLevelRunEmphasis | 0.594(0.062) | 0.639(0.106) | 0.040 |

**Table E8. The averages and standard deviations of the top 20 features in desmoplastic and replacement HGP CRLMs used in tumor area of T1W image**

| NO. | Feature | Desmoplastic | Replacement | p |
| --- | --- | --- | --- | --- |
| 1 | wavelet.HHL_glszm_GrayLevelNonUniformity | 10.242(17.051) | 31.056(48.278) | 0.016 |
| 2 | wavelet.HHH_glrlm_GrayLevelNonUniformityNormalized | 0.499(0.003) | 0.493(0.013) | 0.014 |
| 3 | wavelet.HHH_glcm_Contrast | 0.464(0.027) | 0.486(0.055) | 0.045 |
| 4 | wavelet.HHH_glcm_Idm | 0.769(0.012) | 0.763(0.017) | 0.150 |
| 5 | wavelet.HLH_glszm_GrayLevelNonUniformityNormalized | 0.421(0.133) | 0.350(0.117) | 0.049 |
| 6 | wavelet.HHL_glrlm_GrayLevelNonUniformityNormalized | 0.478(0.052) | 0.459(0.047) | 0.159 |
| 7 | wavelet.HHH_glcm_Id | 0.769(0.052) | 0.764(0.016) | 0.177 |
| 8 | wavelet.HLH_glcm_Correlation | 0.165(0.055) | 0.130(0.071) | 0.039 |
| 9 | wavelet.HHH_firstorder_RootMeanSquared | 2.122(1.966) | 3.448(2.638) | 0.032 |
| 10 | wavelet.HHH_glcm_DifferenceAverage | 0.463(0.025) | 0.476(0.037) | 0.144 |
| 11 | wavelet.HHL_ngtdm_Contrast | 0.056(0.038) | 0.034(0.031) | 0.029 |
| 12 | wavelet.HLH_glrlm_LongRunLowGrayLevelEmphasis | 2.439(1.906) | 1.412(1.207) | 0.032 |
| 13 | wavelet.HHL_firstorder_TotalEnergy | 570254.3(1165657.6) | 1841291.6(3055627.0) | 0.022 |
| 14 | wavelet.HLH_glszm_SmallAreaEmphasis | 0.392(0.184) | 0.464(0.142) | 0.127 |
| 15 | wavelet.HHH_firstorder_Maximum | 15.677(14.960) | 26.924(24.022) | 0.029 |
| 16 | wavelet.LLH_glcm_ClusterProminence | 450.064(1451.967) | 2942.587(5808.554) | 0.012 |
| 17 | wavelet.HHL_glszm_SizeZoneNonUniformity | 9.125(16.852) | 32.189(57.059) | 0.020 |
| 18 | wavelet.HHH_glcm_JointEnergy | 0.255(0.003) | 0.252(0.008) | 0.033 |
| 19 | wavelet.HLH_gldm_LargeDependenceLowGrayLevelEmphasis | 71.075(53.034) | 45.138(39.265) | 0.057 |
| 20 | wavelet.HHH_glcm_Correlation | 0.076(0.048) | 0.062(0.055) | 0.308 |

**Table E9. The averages and standard deviations of the top 20 features in desmoplastic and replacement HGP CRLMs used in tumor area of T2W image**

| NO. | Feature | Desmoplastic | Replacement | p |
| --- | --- | --- | --- | --- |
| 1 | wavelet.LHL_glcm_InverseVariance | 0.411(0.066) | 0.398(0.082) | 0.512 |
| 2 | wavelet.LHL_glcm_Idmn | 0.985(0.008) | 0.981(0.007) | 0.076 |
| 3 | wavelet.HLL_glcm_InverseVariance | 0.408(0.061) | 0.395(0.077) | 0.478 |
| 4 | wavelet.LHL_gldm_DependenceVariance | 12.351(8.817) | 8.108(6.697) | 0.063 |
| 5 | wavelet.HHH_glcm_InverseVariance | 0.498(0.034) | 0.480(0.052) | 0.094 |
| 6 | original_firstorder_10Percentile | 644.960(384.767) | 1071.950(1011.761) | 0.020 |
| 7 | wavelet.LLL_firstorder_10Percentile | 1831.514(1065.365) | 2973.834(2782.137) | 0.024 |
| 8 | wavelet.LHH_glcm_Idmn | 0.979(0.010) | 0.971(0.015) | 0.023 |
| 9 | wavelet.LLL_firstorder_Median | 2464.238(1485.918) | 3773.303(3330.796) | 0.037 |
| 10 | wavelet.LLL_firstorder_Mean | 2471.932(1481.573) | 3747.621(3316.396) | 0.042 |
| 11 | wavelet.LLL_firstorder_RootMeanSquared | 2526.072(1523.156) | 3800.771(3353.091) | 0.045 |
| 12 | original_firstorder_Median | 903.932(541.557) | 1381.525(1217.583) | 0.037 |
| 13 | wavelet.LHH_glrlm_LowGrayLevelRunEmphasis | 0.060(0.072) | 0.135(0.188) | 0.028 |
| 14 | wavelet.LHH_gldm_LowGrayLevelEmphasis | 0.057(0.069) | 0.132(0.187) | 0.027 |
| 15 | wavelet.HLH_glcm_InverseVariance | 0.430(0.079) | 0.392(0.081) | 0.088 |
| 16 | original_firstorder_Minimum | 370.181(308.919) | 625.500(807.043) | 0.079 |
| 17 | original_firstorder_Mean | 932.544(585.660) | 1381.483(1227.855) | 0.057 |
| 18 | wavelet.HHL_glcm_InverseVariance | 0.451(0.025) | 0.457(0.037) | 0.474 |
| 19 | wavelet.LLL_firstorder_Minimum | 1195.534(835.588) | 1897.833(2211.034) | 0.077 |
| 20 | wavelet.LHH_glrlm_LongRunLowGrayLevelEmphasis | 0.275(0.439) | 0.944(1.791) | 0.027 |

**Table E10. The averages and standard deviations of the top 20 features in desmoplastic and replacement HGP CRLMs used in tumor area of arterial phase image**

| NO. | Feature | Desmoplastic | Replacement | p |
| --- | --- | --- | --- | --- |
| 1 | wavelet.HHL_glcm_Correlation | 0.181(0.066) | 0.135(0.062) | 0.011 |
| 2 | wavelet.HHL_gldm_DependenceVariance | 19.760(4.864) | 17.331(4.847) | 0.072 |
| 3 | wavelet.HHL_glcm_InverseVariance | 0.429(0.042) | 0.458(0.027) | 0.007 |
| 4 | wavelet.HHH_glcm_Correlation | 0.027(0.040) | 0.001(0.025) | 0.012 |
| 5 | wavelet.HHH_glcm_Id | 0.749(0.017) | 0.727(0.039) | 0.003 |
| 6 | wavelet.HHH_glcm_Idm | 0.748(0.019) | 0.722(0.047) | 0.003 |
| 7 | wavelet.HLL_glcm_Correlation | 0.460(0.090) | 0.415(0.108) | 0.094 |
| 8 | wavelet.HHH_glcm_InverseVariance | 0.488(0.022) | 0.499(0.013) | 0.039 |
| 9 | wavelet.HHH_glrlm_RunLengthNonUniformityNormalized | 0.417(0.049) | 0.453(0.048) | 0.009 |
| 10 | wavelet.LHL_glcm_InverseVariance | 0.340(0.071) | 0.357(0.099) | 0.143 |
| 11 | wavelet.HHH_glrlm_ShortRunEmphasis | 0.653(0.042) | 0.685(0.042) | 0.007 |
| 12 | wavelet.HHH_firstorder_InterquartileRange | 5.921(4.352) | 10.105(6.250) | 0.003 |
| 13 | wavelet.HHH_gldm_LargeDependenceEmphasis | 150.071(29.201) | 131.601(23.768) | 0.017 |
| 14 | wavelet.LHH_gldm_DependenceVariance | 19.785(4.155) | 18.497(3.899) | 0.251 |
| 15 | wavelet.HHH_firstorder_Uniformity | 0.488(0.017) | 0.464(0.054) | 0.011 |
| 16 | wavelet.HLL_firstorder_Minimum | -297.611(187.955) | -599.959(623.628) | 0.016 |
| 17 | wavelet.HHL_gldm_LargeDependenceEmphasis | 159.980(67.097) | 109.404(52.014) | 0.004 |
| 18 | wavelet.HHH_firstorder_RobustMeanAbsoluteDeviation | 2.589(1.799) | 4.303(2.605) | 0.004 |
| 19 | wavelet.HHL_glrlm_RunLengthNonUniformityNormalized | 0.438(0.131) | 0.532(0.116) | 0.008 |
| 20 | wavelet.HLH_glcm_Correlation | 0.150(0.055) | 0.127(0.062) | 0.152 |

**Table E11. The averages and standard deviations of the top 20 features in desmoplastic and replacement HGP CRLMs used in tumor area of portal venous phase image**

| NO. | Feature | Desmoplastic | Replacement | p |
| --- | --- | --- | --- | --- |
| 1 | wavelet.HHH_glcm_Correlation | 0.045(0.052) | -0.0003(0.027) | 0.001 |
| 2 | wavelet.HHL_glcm_Correlation | 0.196(0.074) | 0.131(0.062) | 0.001 |
| 3 | wavelet.HHH_glcm_InverseVariance | 0.478(0.028) | 0.500(0.013) | 0.002 |
| 4 | wavelet.HHL_glcm_InverseVariance | 0.418(0.041) | 0.462(0.028) | <0.001 |
| 5 | wavelet.HHH_glcm_Id | 0.754(0.022) | 0.728(0.033) | 0.001 |
| 6 | wavelet.HHH_glcm_Idm | 0.753(0.025) | 0.723(0.041) | 0.001 |
| 7 | wavelet.HHL_glcm_JointEnergy | 0.203(0.078) | 0.134(0.068) | 0.001 |
| 8 | wavelet.HHL_gldm_LargeDependenceEmphasis | 171.211(67.422) | 109.164(44.997) | <0.001 |
| 9 | wavelet.HHL_glrlm_GrayLevelNonUniformityNormalized | 0.405(0.106) | 0.315(0.094) | 0.002 |
| 10 | wavelet.HHL_glrlm_ShortRunEmphasis | 0.637(0.116) | 0.741(0.079) | 0.001 |
| 11 | wavelet.HHL_glcm_MaximumProbability | 0.257(0.077) | 0.195(0.069) | 0.003 |
| 12 | wavelet.HHH_firstorder_10Percentile | -6.033(4.932) | -10.678(5.873) | 0.002 |
| 13 | wavelet.HHL_glcm_Id | 0.739(0.096) | 0.666(0.077) | 0.004 |
| 14 | wavelet.HHL_firstorder_Uniformity | 0.422(0.103) | 0.340(0.095) | 0.004 |
| 15 | wavelet.HHL_glcm_Idm | 0.728(0.114) | 0.643(0.093) | 0.005 |
| 16 | wavelet.LHH_glcm_Correlation | 0.190(0.062) | 0.153(0.058) | 0.027 |
| 17 | wavelet.HHH_firstorder_RobustMeanAbsoluteDeviation | 2.217(1.922) | 4.088(2.291) | 0.001 |
| 18 | wavelet.HHH_firstorder_InterquartileRange | 5.121(4.606) | 9.543(5.445) | 0.002 |
| 19 | wavelet.HHL_glrlm_RunPercentage | 0.580(0.118) | 0.680(0.084) | 0.001 |
| 20 | wavelet.HHH_glcm_DifferenceAverage | 0.500(0.058) | 0.576(0.116) | 0.001 |

**Table E12. The averages and standard deviations of the top 20 features in desmoplastic and replacement HGP CRLMs used in tumor area of ADC image**

| NO. | Feature | Desmoplastic | Replacement | p |
| --- | --- | --- | --- | --- |
| 1 | wavelet.HLL_glcm_Imc2 | 0.256(0.096) | 0.313(0.088) | 0.029 |
| 2 | original_glcm_ClusterShade | -0.018(0.052) | -0.054(0.104) | 0.073 |
| 3 | wavelet.HLL_glcm_Correlation | 0.205(0.101) | 0.261(0.093) | 0.043 |
| 4 | wavelet.LLL_gldm_LargeDependenceLowGrayLevelEmphasis | 265.870(156.051) | 230.516(176.513) | 0.429 |
| 5 | original_firstorder_Uniformity | 0.986(0.041) | 0.963(0.070) | 0.121 |
| 6 | original_glcm_ClusterProminence | 0.028(0.079) | 0.088(0.169) | 0.062 |
| 7 | wavelet.LLL_ngtdm_Coarseness | 9024.390(3004.062) | 7500.000(4442.615) | 0.119 |
| 8 | wavelet.LLL_glcm_Imc1 | -0.013(0.043) | -0.037(0.078) | 0.139 |
| 9 | wavelet.LLL_gldm_LowGrayLevelEmphasis | 0.931(0.213) | 0.820(0.320) | 0.112 |
| 10 | wavelet.LLH_glrlm_LongRunLowGrayLevelEmphasis | 7.641(4.138) | 10.590(9.525) | 0.096 |
| 11 | wavelet.HLL_glcm_ClusterProminence | 0.605(0.061) | 0.633(0.053) | 0.083 |
| 12 | wavelet.LLL_glrlm_LowGrayLevelRunEmphasis | 0.936(0.200) | 0.828(0.306) | 0.105 |
| 13 | wavelet.LLL_glszm_GrayLevelNonUniformityNormalized | 0.960(0.126) | 0.890(0.199) | 0.101 |
| 14 | wavelet.LLL_glszm_LowGrayLevelZoneEmphasis | 0.976(0.080) | 0.927(0.140) | 0.085 |
| 15 | wavelet.LLH_glrlm_LongRunEmphasis | 13.169(6.238) | 15.766(9.529) | 0.207 |
| 16 | original_glrlm_GrayLevelNonUniformityNormalized | 0.969(0.080) | 0.936(0.107) | 0.194 |
| 17 | wavelet.LLH_gldm_LargeDependenceHighGrayLevelEmphasis | 313.497(183.993) | 278.126(125.877) | 0.442 |
| 18 | wavelet.LHL_firstorder_10Percentile | -0.0002(<0.001) | -0.0001(<0.001) | 0.014 |
| 19 | wavelet.LLH_firstorder_Skewness | -0.140(0.698) | 0.064(0.591) | 0.265 |
| 20 | wavelet.HLL_ngtdm_Complexity | 0.374(0.054) | 0.353(0.047) | 0.133 |

**Table E13. Akaike information criterion (AIC) of each model in stepwise forward regression.**

| **Models** | **AIC** |
| --- | --- |
| **T2WI** | 180.08 |
| **T2WI + AP** | 154.06 |
| **T2WI + AP + PVP** | 142.57 |
| **T2WI + AP + PVP + ADC** | 143.73 |
| **T2WI + AP + PVP + ADC + T1WI** | 145.53 |

Note: AIC is a standard to measure the goodness of fit of models, and the model with the minimum AIC value is selected

**Table E14. Result of radiomic model that was trained in Hospital 2.**

| **Model** | **Training cohort** | | | | **Internal validation cohort** | | | | **External validation cohort** | | | |
| --- | --- | --- | --- | --- | --- | --- | --- | --- | --- | --- | --- | --- |
|  | **AUC** | **ACC** | **SEN** | **SPE** | **AUC** | **ACC** | **SEN** | **SPE** | **AUC** | **ACC** | **SEN** | **SPE** |
| **T2WI** | 0.758  (0.630-0.886) | 0.617 | 0.524 | 0.833 | 0.763  (0.626-0.901) | 0.733 | 0.579 | 1.000 | 0.681  (0.578-0.783) | 0.674 | 0.661 | 0.700 |
| **AP** | 0.754  (0.628-0.880) | 0.817 | 0.929 | 0.556 | 0.732  (0.578-0.886) | 0.733 | 0.895 | 0.455 | 0.681  (0.574-0.788) | 0.641 | 0.806 | 0.300 |
| **PVP** | 0.735  (0.607-0.863) | 0.833 | 1.000 | 0.444 | 0.763  (0.647-0.879) | 0.700 | 1.00 | 0.182 | 0.753  (0.662-0.844) | 0.652 | 0.952 | 0.333 |
| **Radiomics model** | 0.888  (0.794-0.981) | 0.900 | 1.000 | 0.667 | 0.906  (0.805-1) | 0.767 | 0.947 | 0.455 | 0.788  (0.692-0.884) | 0.761 | 0.790 | 0.700 |

**Table E15. The features that was selected by Hospital 1 and Hospital 2.**

| **Phase** | **Features** | **Description** |
| --- | --- | --- |
| **T2WI** | Wavelet.LLH_glcm_Idmn | Idmn (inverse difference moment normalized) is a measure of the local homogeneity of an image. |
| **AP** | Wavelet.HHL_glcm_Correlation | Correlation shows the linear dependency of gray level values to their respective voxels in the GLCM. |
|  | Wavelet.HLH_firstorder_Skewness | Skewness measures the symmetry of the distribution of gray values. |
| **PVP** | Wavelet.HHL_glcm_InverseVariance | Inverse variance is the reciprocal of variance. |

## Supplementary Figures


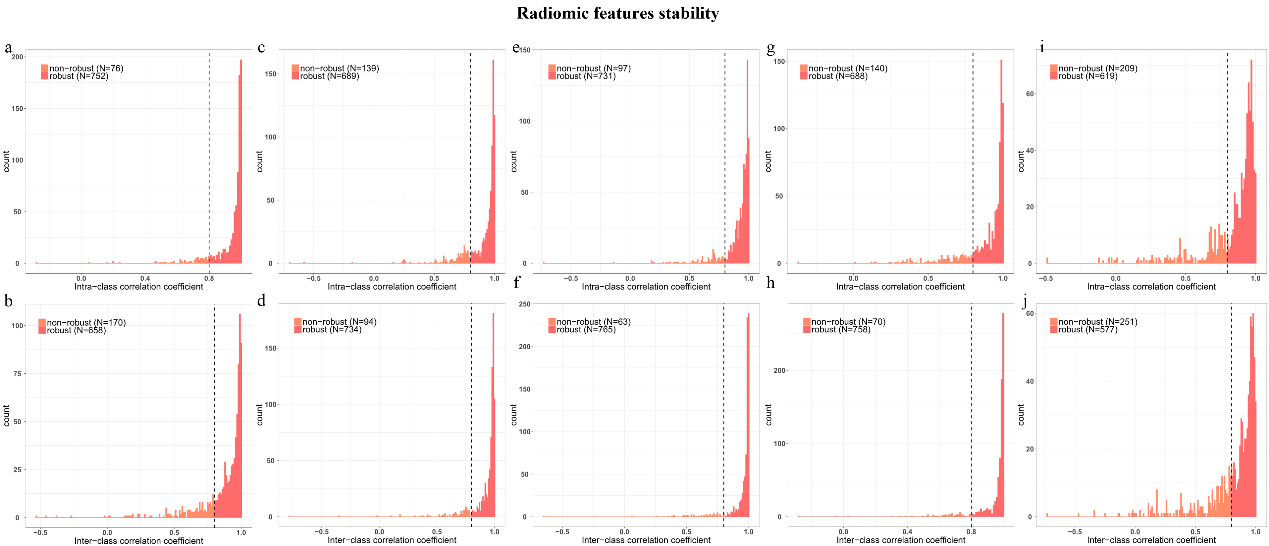


**Figure E1.** Intra-/inter-class correlation coefficient of radiomic features extracted from TLI area. (a) and (b): radiomic features in T1W images; (c) and (d): radiomic features in T2W images; (e) and (f): radiomic features in arterial phase images; (g) and (h): radiomic features in portal venous phase images; (i) and (j): radiomic features in ADC images.

**
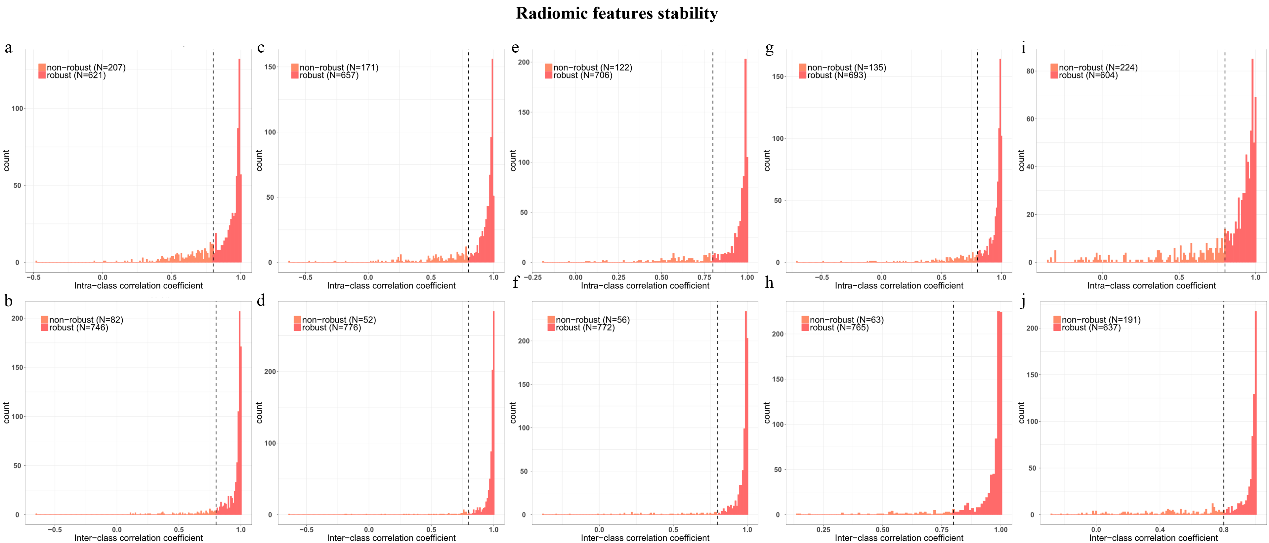
**

**Figure E2** Intra-/inter-class correlation coefficient of radiomic features extracted from tumor area. (a) and (b): radiomic features in T1W images; (c) and (d): radiomic features in T2W images; (e) and (f): radiomic features in arterial phase images; (g) and (h): radiomic features in portal venous phase images; (i) and (j): radiomic features in ADC images.


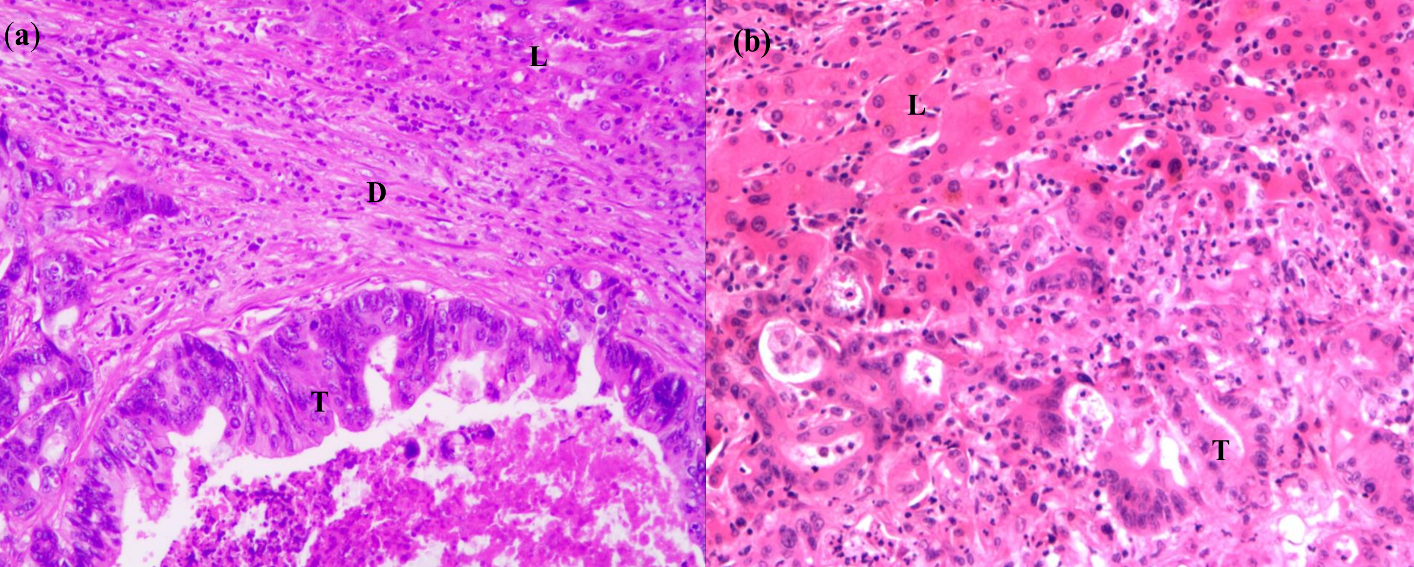


**Figure E3** H&E staining images of CRLMs with different HGPs. (a) High magnification （×200）images of the desmoplastic growth pattern. (b) High magnification （ ×200 ） images of the replacement growth pattern. T, tumor tissue; L, liver parenchyma; D, dense fibrous band.


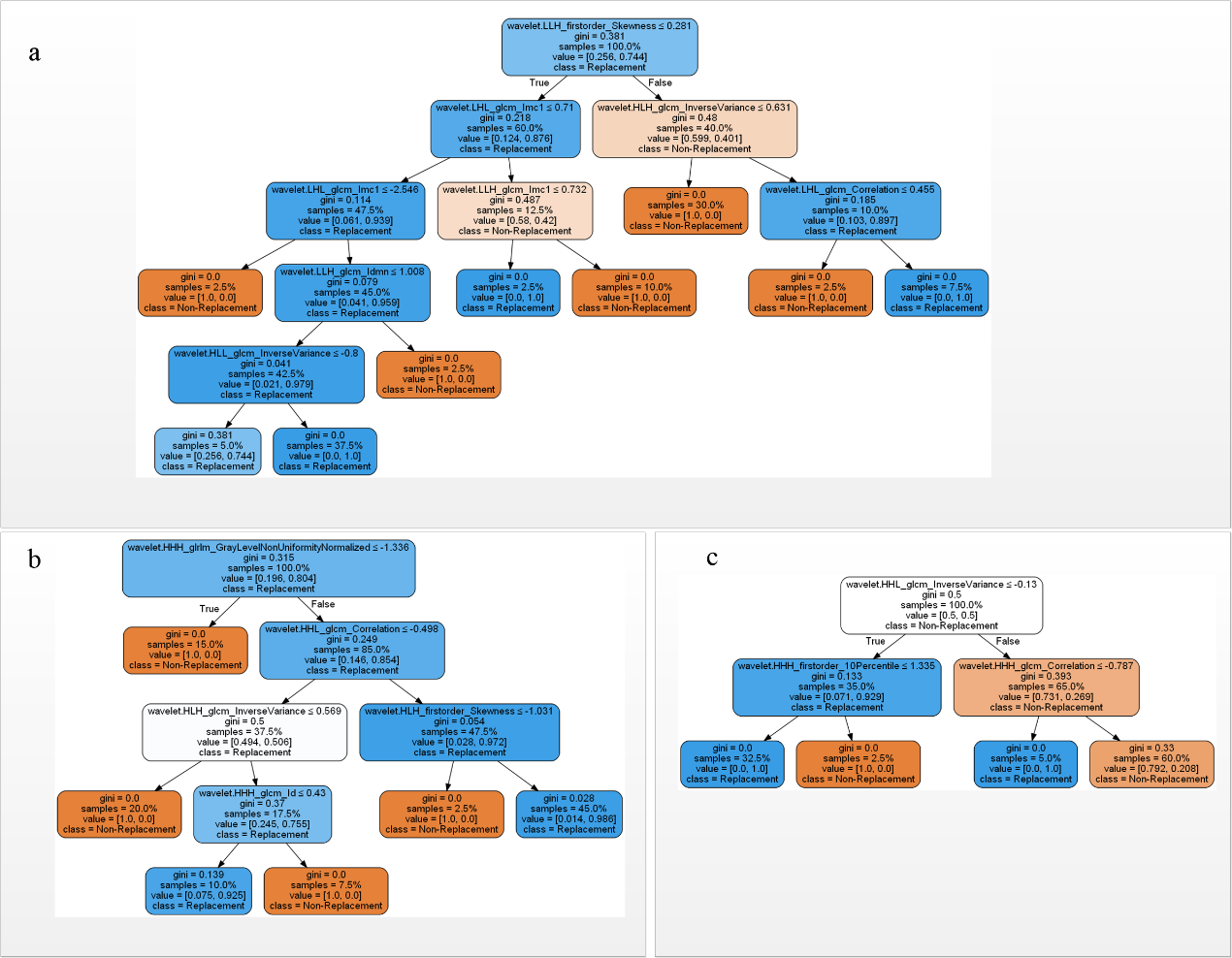


**Figure E4** (a) Decision tree of T2W image; (b) decision tree of arterial phase image; (c) decision tree of portal venous phase image.


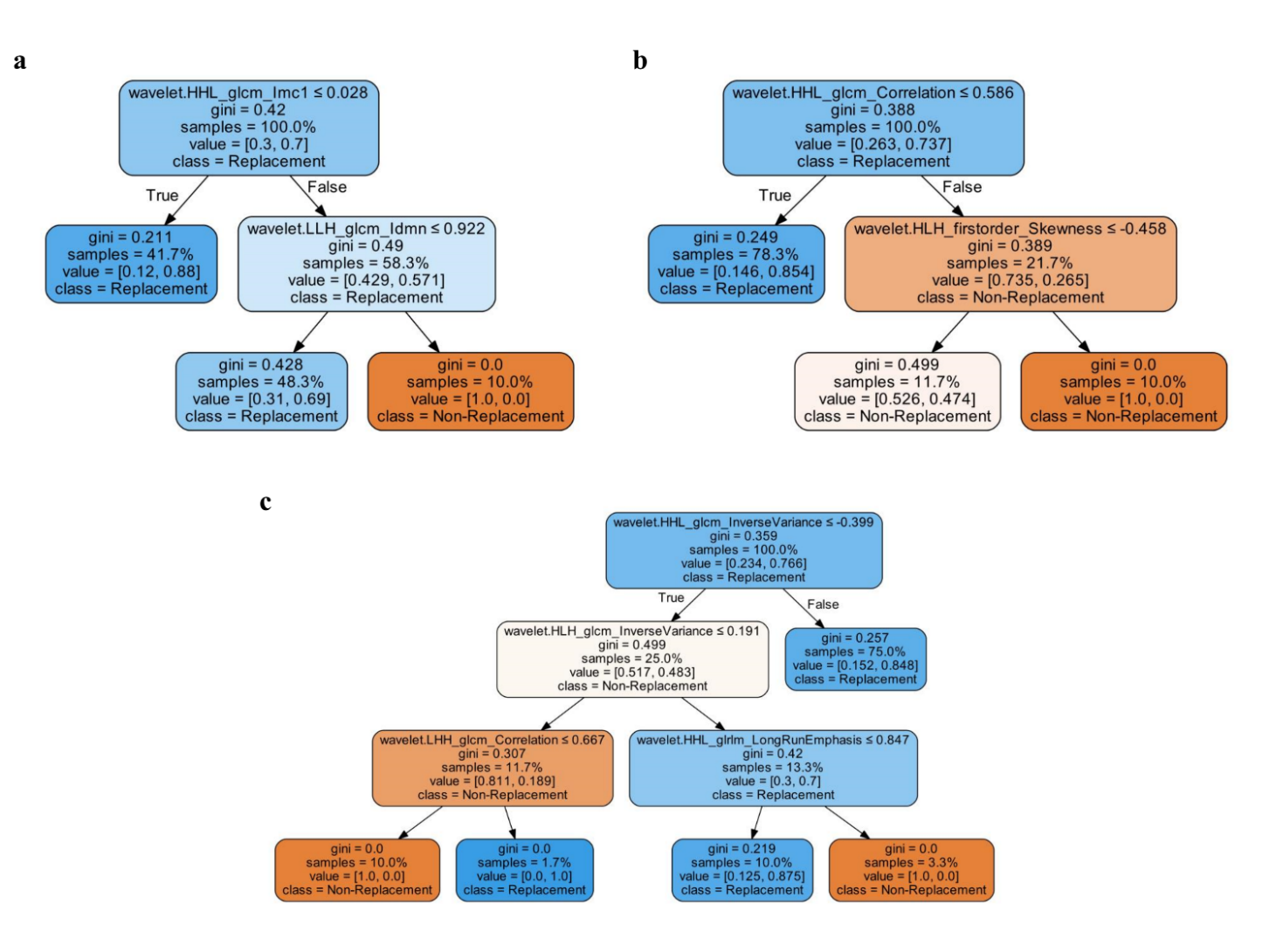


**Figure E5** The decision tree that was build in Hospital 2. (a) Decision tree of T2W image; (b) decision tree of arterial phase image; (c) decision tree of portal venous phase image.

**Supplementary References**

1. Haralick RM, Shanmugam K, Dinstein I. Textural features for image classification. *IEEE Transactions on Systems, Man and Cybernetics*. (1973) SMC-3; SMC-3: 610-21. doi:10.1109/TSMC.1973.4309314

2. Galloway MM. Texture analysis using gray level run lengths. *Computer Graphics and Image Processing*. (1975) 4: 172-9. doi:https://doi.org/10.1016/S0146-664X(75)80008-6

3. Chu A, Sehgal CM, Greenleaf JF. Use of gray value distribution of run lengths for texture analysis. *Pattern Recogn Lett*. (1990) 11: 415-9. doi:https://doi.org/10.1016/0167-8655(90)90112-F

4. Dasarathy BV, Holder EB. Image characterizations based on joint gray level—run length distributions. *Pattern Recogn Lett*. (1991) 12: 497-502. doi:https://doi.org/10.1016/0167-8655(91)80014-2

5. Thibault G, Fertil B, Navarro C, Pereira S, Cau P, Levy N, et al. Texture indexes and gray level size zone matrix application to cell nuclei classification. In: *10th International Conference on Pattern Recognition and Information Processing*., (2009)

6. Amadasun M, King R. Textural features corresponding to textural properties. *IEEE Transactions on Systems, Man and Cybernetics*. (1989) 19; 19: 1264-74. doi:10.1109/21.44046
